# Genetic regulation of *AIF1* shapes immune and liver injury profiles in chronic alcohol use

**DOI:** 10.1172/jci.insight.198209

**Published:** 2026-03-10

**Authors:** Priscila C. Antonello, Colin A. Hodgkinson, Dechun Feng, Cheryl Marietta, Baskar Mohana Krishnan, Maria A. Parra, Zhaoli Sun, Bin Gao, David Goldman, Michelle W. Antoine

**Affiliations:** 1 Section on Neural Circuits,; 2 Laboratory of Neurogenetics, and; 3 Laboratory of Liver Diseases, National Institute on Alcohol Abuse and Alcoholism, NIH, Bethesda, Maryland, USA.; 4 Transplant Biology Research Center, Johns Hopkins University School of Medicine, Baltimore, Maryland, USA.

**Keywords:** Genetics, Hepatology, Public Health, Addiction, Biomarkers, Genetic risk factors

## Abstract

**BACKGROUND:**

In chronic alcohol consumers, immune cells may drive the progression from mild liver injury to more severe alcohol-associated liver disease (ALD), including alcohol-associated hepatitis (AAH) and cancer. Liver macrophages, both resident and infiltrating, express allograft inflammatory factor 1 (AIF1), which is upregulated during inflammation and enhances immune activation.

**METHODS:**

Using serum and urine samples from 868 individuals classified as having alcohol use disorder or not, based on DSM-IV/V criteria, along with serum and liver biopsy tissue from a second cohort of 27 patients diagnosed with AAH, we evaluated the impact of the *AIF1* promoter single-nucleotide polymorphism (SNP) (rs3132451; *C/C*, *C/G*, *G/G*) on liver function markers and immune cell profiles.

**RESULTS:**

*AIF1* transcript levels were genotype dependent: *C/C* homozygotes expressed 5.2% of the levels observed in *G/G* individuals, while *C/G* heterozygotes expressed 46%. Unlike most SNPs associated with harmful effects, the *G/G* genotype is highly prevalent, present in about 70% of patients. Among chronic alcohol users, *G/G* individuals exhibited elevated markers of liver injury and a more than 3-fold increase in hepatic immune cells, including infiltrating AIF1^+^ macrophages and neutrophils. Despite similar durations of alcohol misuse, *G/G* individuals had higher Model for End-Stage Liver Disease scores compared with *C/G* individuals, indicating a significantly greater 90-day mortality risk. Notably, some immune abnormalities, such as elevated neutrophils, persisted in *G/G* males even after alcohol abstinence.

**CONCLUSION:**

These findings suggest that functional genetic variation in *AIF1* may contribute to the severity and persistence of ALD.

**TRIAL REGISTRATION:**

ClinicalTrials.gov NCT02231840.

**FUNDING:**

Research support was provided from the National Institute on Alcohol Abuse and Alcoholism of the NIH under grants 1ZIAAA000440-02 and R24AA025017.

## Abstract 2

A common SNP in the AIF1 gene exacerbates liver injury severity in chronic alcohol users, with G/G individuals showing increased inflammation, liver damage, and mortality risk

## Graphical abstract

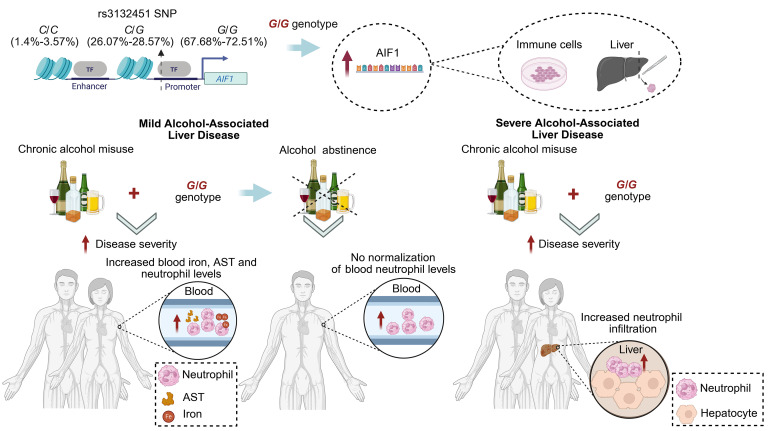

## Introduction

Alcohol use disorder (AUD) is characterized by a chronic, compulsive urge to consume alcohol despite its detrimental consequences on health and daily life (1). Prolonged and excessive alcohol consumption can cause varying degrees of liver damage, collectively known as alcohol-associated liver disease (ALD) (2). The earliest stage of ALD is fatty liver, or alcohol-associated steatosis, characterized by fat accumulation in liver cells. This condition affects over 90% of heavy drinkers (those consuming more than 60 g/d) (3) and is reversible with alcohol cessation. However, continued heavy drinking leads to progression in approximately 3%–35% of individuals, resulting in more severe forms of ALD such as alcohol-associated hepatitis (AAH), alcohol-associated cirrhosis (AC), and liver cancer — potentially culminating in liver failure.

While multiple risk factors may contribute to the progression of ALD, chronic liver inflammation is thought to play a major role, with approximately 35% of heavy drinkers developing persistent inflammation (2–5). Unlike acute inflammation, which is typically protective and self-limited, chronic inflammation can lead to sustained liver injury. Although the precise mechanisms remain incompletely understood, prolonged alcohol exposure is thought to trigger immune cell recruitment and activate molecular signaling pathways that drive gene expression changes in these cells. Several immune cell types contribute to alcohol-induced inflammation, including resident liver macrophages (Kupffer cells) and infiltrating cells such as neutrophils, monocyte-derived macrophages, and T and B lymphocytes (4, 5). This immune cell activation promotes the release of cytokines, chemokines, and other proinflammatory mediators that, rather than facilitating tissue repair, exacerbate liver injury and impair function (4).

The gene allograft inflammatory factor 1 (*AIF1*) encodes a calcium-binding protein associated with inflammation and immune responses (6). It is highly expressed (>1,500–3,300 transcripts per million) in myeloid-lineage immune cells, including monocytes, all tissue-resident macrophages, and specialized subsets such as Kupffer cells in the liver and microglia in the brain (7–13). In these immune cell types — particularly macrophages — *AIF1* enhances critical immune functions, including cell proliferation, migration, and activation (10–13). Once activated, tissue-resident macrophages recruit neutrophils to the sites of inflammation by releasing proinflammatory cytokines and chemoattractants (14–17). Thus, *AIF1* expression plays a crucial role in promoting coordinated crosstalk between neutrophils and macrophages during infection and following tissue injury (18).

Neutrophils, the most abundant type of white blood cell in humans, are readily recruited to areas of infection or tissue injury (19). They are key contributors to host defense through the production of reactive oxygen species (ROS), including free radicals such as superoxide anions and hypochlorous acid, during a process known as the respiratory or oxidative burst to eliminate pathogens (20). In addition, neutrophils phagocytose pathogens and infected cells and secrete proinflammatory cytokines that initiate inflammation and recruit other immune cells. However, the cytotoxic potential of ROS necessitates tight regulation of neutrophil activation and their rapid clearance during inflammation to avoid collateral tissue damage. Recent studies have implicated neutrophils as key contributors to chronic inflammation, particularly when their recruitment becomes continuous (19).

In addition to metabolizing toxins and alcohol, the liver plays a central role in regulating iron homeostasis. Altered iron levels are a key feature of ALD, as excess iron contributes to oxidative stress and hepatocellular injury, potentially worsening inflammation (21). The liver regulates iron balance primarily through the hepatic production of the hormone hepcidin, which inhibits iron absorption in the duodenum, promotes iron sequestration by macrophages, and limits iron storage within hepatocytes (22). Hepcidin deficiency, whether due to genetic deletion in mice or mutations in humans, results in iron overload and iron accumulation in the liver (22, 23). Notably, approximately 60%–64% of ALD cases exhibit reduced serum hepcidin levels (24), along with elevated serum markers of iron overload, including increased transferrin saturation, ferritin, and hepatic iron concentration (24, 25).

Iron also plays a key role in neutrophil function, including promoting ROS production and phagocytosis (26). Experimental hypoferremia (low plasma iron) induced by hepcidin treatment in mice reduced ROS generation, reduced bactericidal activity against *Escherichia coli* and *Staphylococcus aureus*, and suppressed *CCL2* and *TNF-α* cytokine production in neutrophils following ex vivo stimulation with zymosan particles (27). Similarly, iron deficiency anemia impairs the oxidative burst in humans (27–29), while iron overload in hereditary hemochromatosis enhances it (29). Therefore, iron overload may promote neutrophil hyperactivity and exacerbate tissue injury. Taken together, these findings suggest that elevated iron markers not only indicate disrupted hepatic iron regulation but may also serve as indicators of neutrophil function or integrity.

Genomic studies, including genome-wide association studies (GWAS), have implicated *AIF1* as a significant locus in various inflammatory diseases, namely rheumatoid arthritis, multiple sclerosis, and inflammatory bowel disease (30). According to the Genome Aggregation Database (gnomAD; v4, Broad Institute) (31), several single-nucleotide polymorphisms (SNPs) occur in *AIF1*. While most are synonymous or non-coding and are expected to have no functional effect, 7 extremely rare variants — each with allele frequencies ranging from 6.84 × 10^–7^ to 3.42 × 10^–6^ — are predicted to be loss-of-function missense mutations. In disease, elevated, rather than reduced, numbers of *AIF1^+^* cells have been reported in several inflammatory conditions, including multiple sclerosis (32), rheumatoid arthritis (33), and lupus (34, 35), where *AIF1* is presumed to contribute to inflammatory pathogenesis. Thus far, *AIF1* has not been observed to be a factor in ALD. However, genomic studies of ALD performed to date have been small and underpowered, detecting few of the genes contributing to ALD. For AAH, genes involved in inflammation were, as a group, strongly implicated, but largely by genes that at this point are not exome-wide significant (36).

In this study, we investigated how genetic risk and alcohol consumption contribute to ALD. Using serum and urine samples from 868 individuals classified as either having current AUD (CAUD) or not (non-AUD) based on DSM-IV/V criteria, we examined the impact of the only known SNP in the *AIF1* promoter region (rs3132451; *C/C*, *C/G*, or *G/G*) on liver function and immune system integrity. We also explored the functional significance of this *AIF1* variant, as it may be relevant to any disease in which *AIF1* has been implicated. Because promoters are critical regulatory elements that govern when, where, and how much a gene is transcribed, SNPs in promoter regions are often functionally significant. As our patient cohort did not include individuals with advanced ALD diagnoses such as AAH and AC, it allowed us to focus specifically on the role of *AIF1* genotype and alcohol use in the early stages of liver disease. To further evaluate the impact of *AIF1* genotype on AUD-related symptoms and liver function, we analyzed a second cohort of patients diagnosed with AAH, a condition that, when severe, can lead to rapid liver failure and high short-term mortality.

We found that *G/G* homozygous individuals exhibited the highest *AIF1* transcript levels in both immune cells and liver tissue. Regardless of genotype, chronic alcohol use was associated with elevated serum levels of myeloid-lineage immune cells, specifically monocytes and neutrophils. Liver injury biomarkers — γ-glutamyl transferase (GGT), alanine aminotransferase (ALT), and aspartate aminotransferase (AST) — were also elevated in these individuals. While the *G/G* genotype alone did not independently predict these changes, its interaction with chronic alcohol misuse significantly exacerbated AUD-related effects. Specifically, AUD individuals with the *G/G* genotype were more likely to exceed clinical thresholds for conditions such as neutrophilia (elevated neutrophil counts) and iron overload. In AUD patients with AAH, those carrying the *G/G* genotype exhibited significantly greater numbers of hepatic immune cells — including infiltrating *AIF1^+^* macrophages and neutrophils — as well as higher Model for End-Stage Liver Disease (MELD) scores, a composite measure used to assess liver function and predict mortality. These findings support a link between the *AIF1* genotype and advanced disease progression. In a separate cohort of abstinent AUD patients, elevations in immune cell populations, particularly monocytes and neutrophils, persisted despite abstinence, suggesting that some immune alterations may require several years to fully resolve. Notably, the *G/G* genotype — especially in males — was associated with persistent neutrophilia. Together, these findings suggest that functional genetic variation in *AIF1* may modulate the severity of alcohol-induced immune dysregulation and liver injury, primarily by affecting neutrophil biology during both early and advanced stages of ALD.

## Results

### The rs3132451 G/G genotype predicts higher AIF1 expression.

**Figure 1:**
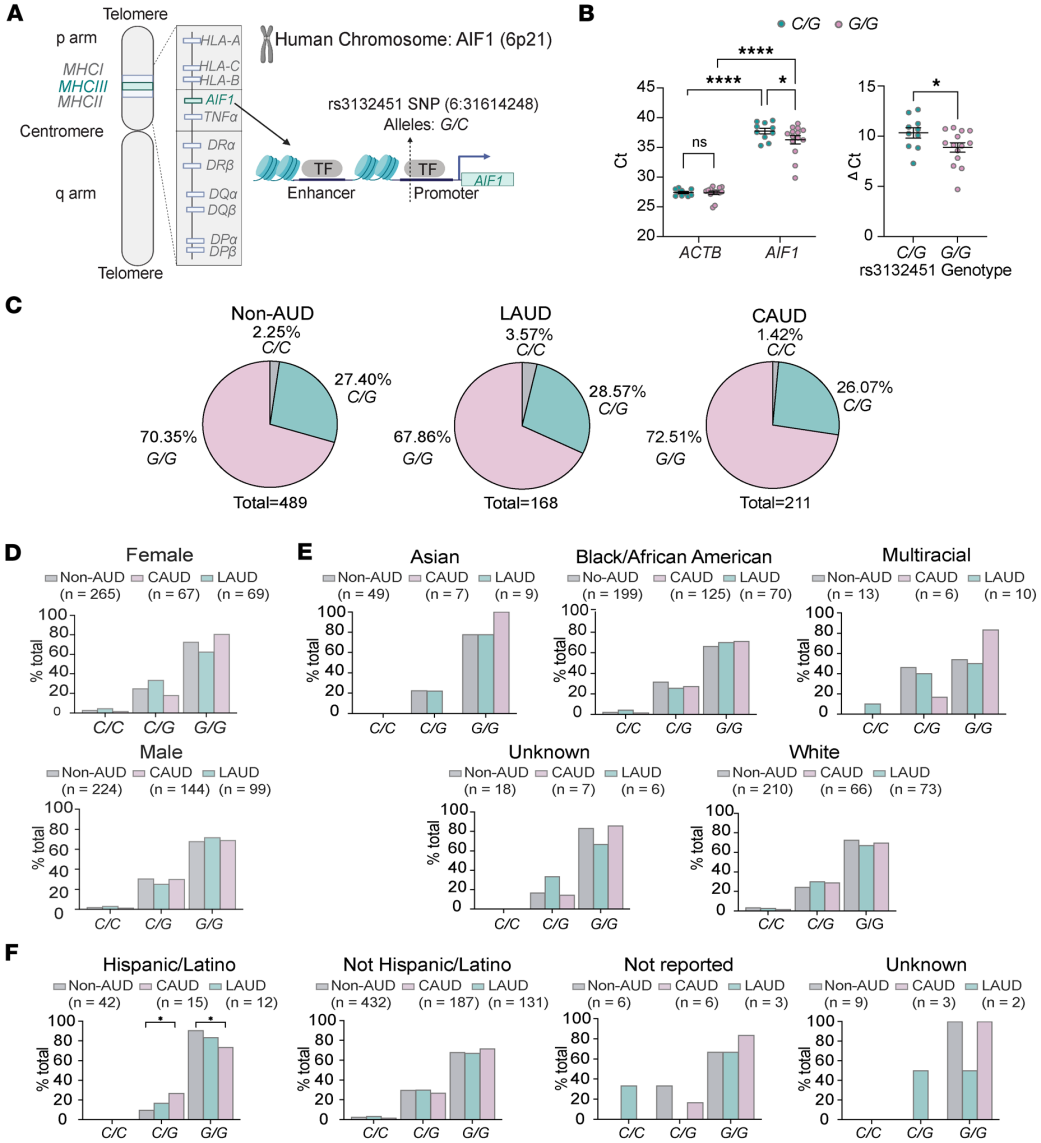
The rs3132451 *G/G* genotype is associated with markedly higher AIF1 expression. (**A**) Schematic of the *AIF1* gene locus on human chromosome 6p21.33 with flanking genes. Inset shows the location of the rs3132451 SNP (alleles G or C) in the promoter region of the *AIF1* gene. (**B**) Scatter dot plot showing cycle threshold (Ct) values obtained by quantitative reverse transcription PCR for *AIF1* and *ACTB* RNA in cDNA from lymphoblastoid cell lines of individuals with rs3132451 *C/G* and *G/G* genotypes. Statistical analysis using a mixed-effects model with Fisher’s LSD post hoc test revealed a significant main effect of SNP genotype (*P* < 0.0001). Comparison of Ct values between *ACTB* and *AIF1* showed a significant difference (*P* < 0.0001). ΔCt values are relative to *ACTB*. A 2-tailed *t* test comparing AIF1 expression between *C/G* and *G/G* genotypes yielded a significant difference (*P* = 0.027). Each point represents an individual sample; data are shown as the mean ± SEM. *P < 0.05, ****P < 0.0001. (**C**) The distribution of *C/C*, *C/G*, and *G/G* genotypes among individuals without a diagnosis of alcohol use disorder (non-AUD), with current AUD (CAUD; per DSM-IV/V criteria), and with a lifetime history of AUD but not meeting criteria within the past year (LAUD). Percentages were compared using Fisher’s exact test (α = 0.05). (**D**) Genotype prevalence by sex across non-AUD, CAUD, and LAUD groups. Statistical analysis was performed using a 2-tailed binomial test (α = 0.05). (**E**) Same as **D**, stratified by race. All *P* values > 0.05. (**F**) Same as **D**, stratified by ethnicity. Statistical analysis was performed using a 2-tailed binomial test (α = 0.05). *P* = 0.0478 for comparisons of *C/G* and *G/G* genotype frequencies between non-AUD and CAUD participants who self-identified as Hispanic/Latino.

The rs3132451 *G/C* polymorphism is located in the promoter region of *AIF1* (Figure 1A). To determine whether this SNP predicts *AIF1* gene expression, we quantified *AIF1* RNA transcripts from lymphoblastoid cell lines derived from blood lymphoblasts of study participants. Of the 24 lymphoblastoid cell lines genotyped for rs3132451, only one was *C/C*, consistent with the low frequency of the *C* allele in the general population, where the average allele frequency is estimated at 2.3% (40). Consequently, *AIF1* RNA expression was quantified only in *C/G* and *G/G* patient cell lines using quantitative reverse transcription PCR and with β-actin (*ACTB*) serving as the control. The cycle threshold (Ct) values for *ACTB* were similar across genotypes, but genotype differences were observed for *AIF1* (Figure 1B). Specifically, Ct values for *AIF1* were significantly higher in *C/G* samples compared with *G/G* samples (Figure 1B), indicating higher *AIF1* expression in the *G/G* genotype. Fold-change analysis based on average ΔCt values, normalized to *ACTB*, showed that *AIF1* expression in *C/G* cells was reduced to 36% of that in *G/G* cells (Figure 1B). *AIF1* RNA expression was also quantified in liver tissue from 29 patients with advanced ALD (2 *C/C*, 10 *C/G*, and 17 *G/G*). The observed genotype frequencies (2/29 *C/C* [6.90%], 10/29 *C/G* [34.48%], and 17/29 *G/G* [58.62%]) fall within the expected range of population-level variation shown in Figure 1A (1.4%–3.57% *C/C*, 26.07%–28.57% *C/G*, and 67.68%–72.51% *G/G*) and are not statistically significant (Supplemental Table 1; supplemental material available online with this article; https://doi.org/10.1172/jci.insight.198209DS1). Fold-change analysis based on average ΔCt values (*C/C* = 20.39, *C/G* = 17.43, and *G/G* = 16.14) again revealed a significant genotype-dependent effect (*P* < 0.035, *F* = 4.061, degrees of freedom [numerator] = 2, and degrees of freedom [denominator] = 17.82, Brown-Forsythe ANOVA test). *AIF1* expression in *C/C* homozygotes was only 5.2% of that observed in *G/G* homozygotes, while *C/G* heterozygotes expressed 46%, consistent with a gene dosage effect (*P* = 0.0007, Dunnett’s T3 multiple-comparison test).

Given that upregulation of *AIF1* is predicted to promote inflammation (8, 10–13, 37, 38), we investigated whether *G/G* AUD patients who chronically consumed excessive amounts of alcohol were more likely to experience adverse health outcomes compared with *C/C* or *C/G* patients. We analyzed 868 individuals based on their rs3132451 genotypes (*C/C*, *C/G*, and *G/G*) and current alcohol consumption status (Figure 1C), categorizing them as either currently meeting DSM-IV and DSM-V criteria for AUD (CAUD) or not (non-AUD). Patients who had not met criteria for AUD within the past year, similar to being in recovery, were classified as long-term abstinent AUD (LAUD). Demographic characteristics of the AUD patient cohort are summarized in Table 1.

Population-level genotype frequencies predict that 75.1% of individuals have the *G/G* genotype and 22.6% are *C/G* heterozygotes (39), closely matching the genotype frequencies we observed. No statistically significant differences in allele frequencies were observed between AUD and LAUD patients compared with non-AUD controls (Figure 1C), consistent with prior large-scale GWAS studies showing that *AIF1* is not associated with AUD or increased drinking behavior (40–42). We further assessed the genotype distribution across sex, race, and ethnicity (Figure 1, D–F). Genotype proportions were similar between males and females and among racial groups. However, among AUD patients who self-identified as Hispanic/Latino, we observed a reduced prevalence of *C/G* heterozygotes, consistent with gnomAD data showing ethnic variation in allele frequency (31).

### Individuals with AUD exhibit higher monocyte and neutrophil counts, elevated serum iron levels, and increased serum levels of several liver enzymes.

**Figure 2:**
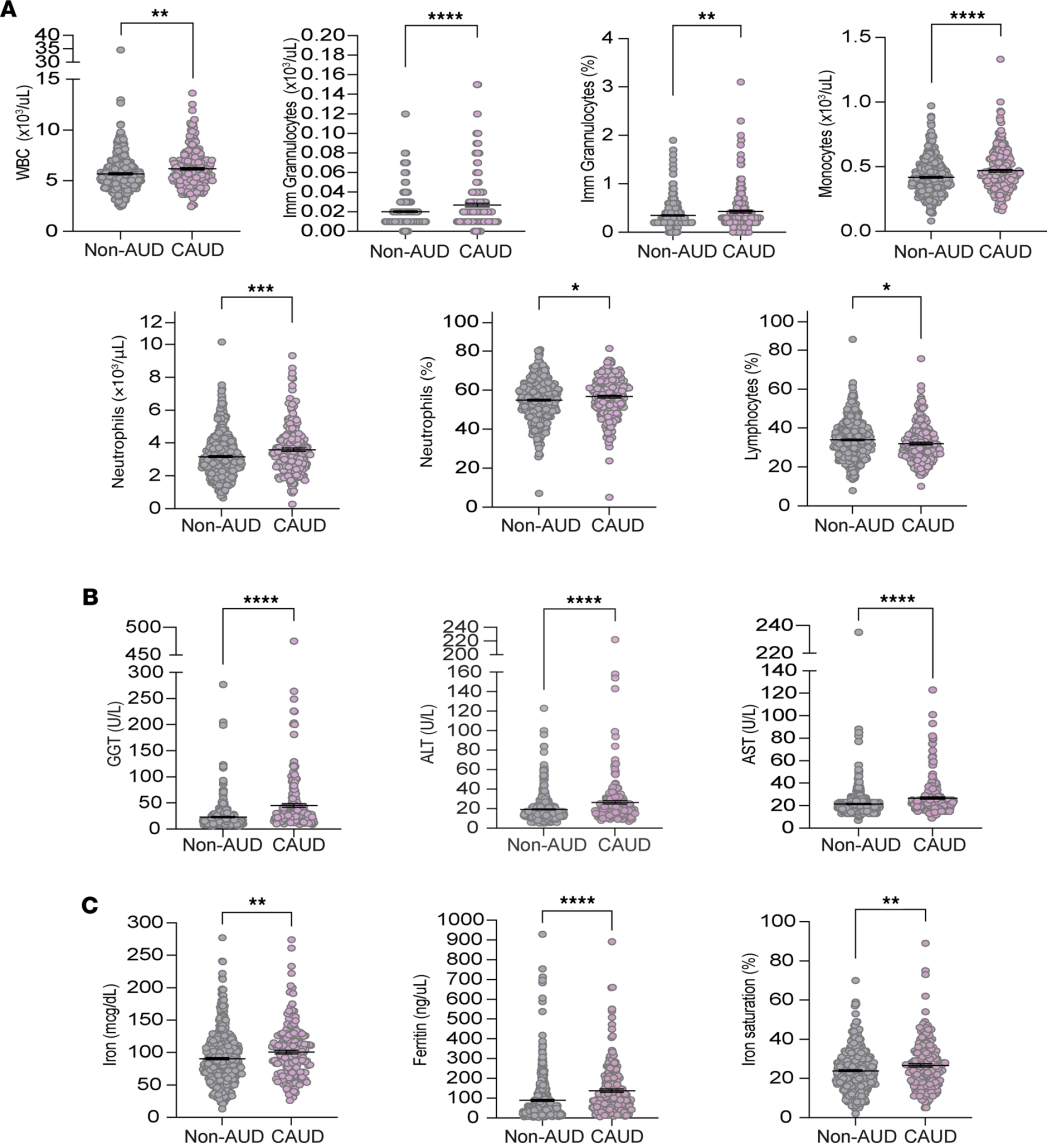
Individuals with AUD exhibit higher monocyte and neutrophil counts, elevated iron levels, and increased levels of several liver enzymes. (**A**) Elevated immune cell populations are observed in individuals with AUD. Imm, immature. (**B**) Iron levels and iron metabolism biomarkers elevated in individuals with AUD. (**C**) Liver function biomarkers altered by AUD. Each point represents an individual sample; data are shown as the mean ± SEM. Statistical comparisons were made between individuals without a diagnosis of AUD (non-AUD) and those with current AUD (CAUD; defined by DSM-IV/V criteria) using an unpaired 2-tailed Student’s *t* test (α = 0.05). *P < 0.05, **P < 0.01, ***P < 0.001, ****P < 0.0001.

To investigate how alcohol consumption and genetic variation at the *AIF1* SNP may contribute to ALD, we first examined the effect of each factor independently, beginning with alcohol consumption. We analyzed serum and urine samples from non-AUD and CAUD patients to assess biomarkers for liver function, immune system integrity, and iron metabolism. Of the 26 biomarkers analyzed, we found that 43% (3/7) related to liver function, 47% (7/15) related to immune system integrity, and 75% (3/4) related to iron metabolism were altered in the CAUD group. Compared with the non-AUD group, CAUD patients showed an increase in the percentages of total white blood cells (WBCs) (Figure 2A). This increase was largely attributed to elevations in specific subtypes of WBCs, namely immature granulocytes, monocytes, and neutrophils (Figure 2A). Immature granulocytes are early forms of the 3 main types of granulocytes: neutrophils, eosinophils, and basophils. However, eosinophil and basophil levels were unaffected (eosinophils: non-AUD, 0.14 ± 0.01; CAUD, 0.14 ± 0.007; *P* = 0.9640, *t* = 0.04, df = 338; basophils: non-AUD, 0.04 ± 0.0009; CAUD, 0.04 ± 0.001; *P* = 0.31, *t* = 1.01, df = 698). Elevations in WBCs, particularly neutrophils and monocytes, indicate ongoing activation of the immune system (43, 44).

Previous studies in adult mice have shown that chronic alcohol consumption can significantly decrease peripheral B cell counts and impair their function (45, 46). Consistent with this, we observed that the percentage of lymphocytes (B cells and T cells) was decreased in the CAUD group compared with the non-AUD group (Figure 2A). Overall, our findings suggest that chronic excessive alcohol use may suppress adaptive immune cells while promoting the expansion of myeloid-lineage phagocytes — monocytes and neutrophils — key components of the innate immune response to infection and injury.

Examination of biomarkers associated with liver function revealed elevated serum concentrations of 3 liver enzymes — GGT, ALT, and AST — in the CAUD group but not in the non-AUD group (Figure 2B). As previously reported, about 60%–64% of ALD cases exhibit elevated serum iron indices (e.g., transferrin saturation, ferritin) and increased hepatic iron concentration (24, 25). Consistent with these findings, we observed elevated serum concentrations of iron and ferritin, as well as increased iron saturation, in the CAUD group, collectively indicating higher systemic iron levels in patients with AUD (Figure 2C). Overall, our findings suggest that AUD is associated with increased monocyte and neutrophil counts and significant adverse effects on liver function.

### G/G patients with CAUD exhibit elevated serological markers of hepatic stress and injury.

**Figure 3:**
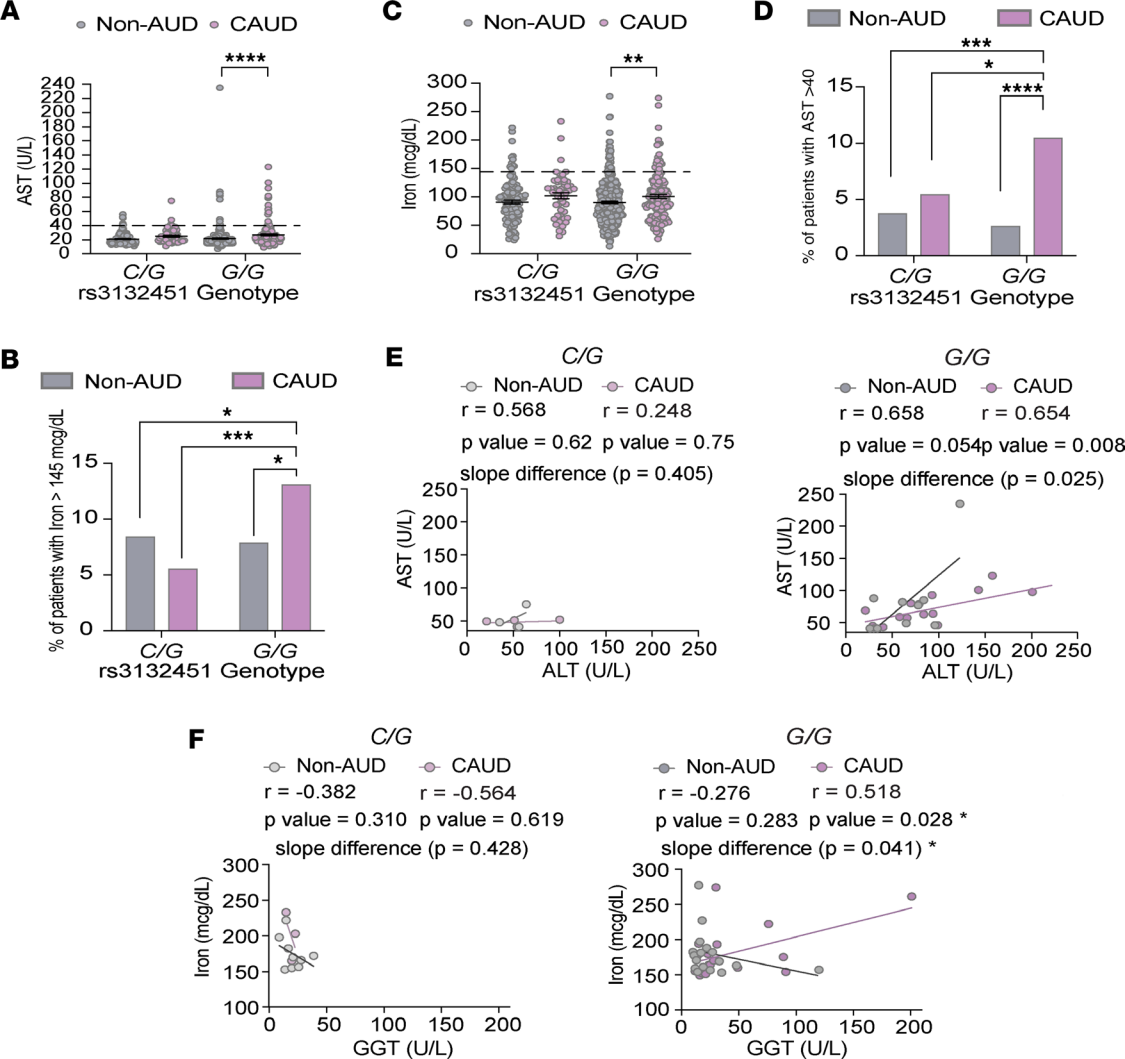
*G/G* patients with CAUD exhibit elevated serological markers of hepatic stress and injury. (**A**) Scatter dot plot showing aspartate aminotransferase (AST) levels in patients with *C/G* and *G/G* genotypes across non-AUD and CAUD groups. Each point represents an individual patient; data are shown as the mean ± SEM. The dashed line marks the upper limit of normal levels. Statistical analysis was performed using 2-way ANOVA with Fisher’s LSD post hoc test with ****P < 0.0001. (**B**) Percentage of patients with AST levels exceeding the upper limit of normal across *C/G* and *G/G* genotypes in non-AUD and CAUD groups. Statistical comparisons were made using a 2-tailed binomial test with **P < 0.01. (**C**) Same as **A**, but for serum iron levels in patients with *C/G* and *G/G* genotypes. *P < 0.05, ***P < 0.001, ****P < 0.0001. (**D**) Same as **B**, but for serum iron levels exceeding the upper limit of normal. *P < 0.05, ***P < 0.001. (**E**) Deming linear regression showing the relationship between elevated AST levels (above the upper limit of normal) and alanine aminotransferase (ALT) in patients with *C/G* and *G/G* genotypes across non-AUD and CAUD groups. Pearson’s *r* and *P* values are reported; the *P* value for the slope was determined by *F* test. Each point represents an individual patient. (**F**) Same as **E**, but for the relationship between elevated iron levels and γ-glutamyl transferase (GGT).

To investigate how genetic variation at the *AIF1* SNP may contribute to ALD, we analyzed the non-AUD and AUD groups by genotype only (*G/G*, *C/G*, *C/C*). None of the 13 biomarkers examining immune response (Figure 2A), liver injury (Figure 2B), and iron levels (Figure 2C) showed statistical significance across genotypes (Supplemental Figure 1). Given that *AIF1* activation requires a stimulus — such as tissue injury, inflammatory cell activation, or cytokine release — it is not surprising that genotype alone had no effect. However, when assessing the combined influence of alcohol consumption and genetics, we observed genotype-specific effects among *G/G* individuals: specifically, *G/G* AUD cases showed significantly elevated AST and iron levels compared with *G/G* non-AUD individuals (Figure 3, A and B). For both biomarkers, the likelihood of exceeding the upper limit of normal — AST greater than 40 U/L and iron greater than 145 μg/dL — was significantly higher in *G/G* AUD patients than in other genotype groups (Figure 3, C and D). Since elevated AST can also reflect heart or muscle damage, we examined whether AST greater than 40 U/L co-occurred with elevations in ALT and GGT, markers more specific to liver injury. In *G/G* AUD patients, elevated AST levels were significantly positively correlated with ALT levels (Figure 3E), but not with GGT (AST-GGT correlation: *r* = 0.09, *P* = 0.769). This pattern was not observed in *C/G* AUD individuals (Figure 3E).

Given that excess iron can induce oxidative liver damage, we further investigated whether elevated iron levels co-occurred with GGT or AST elevations. Notably, iron levels above the normal range were strongly correlated with elevated GGT in the *G/G* AUD group, but not in the *C/G* AUD group (Figure 3F). No significant correlations were found between iron and AST in any group. Together, these findings suggest that *G/G* patients with AUD may exhibit more pronounced serological indicators of hepatic stress or injury, particularly reflected by stronger associations between elevated AST and ALT, as well as between iron and GGT.

### The rs3132451 G/G SNP is associated with elevated neutrophil counts in both blood and liver in ALD.

**Figure 4:**
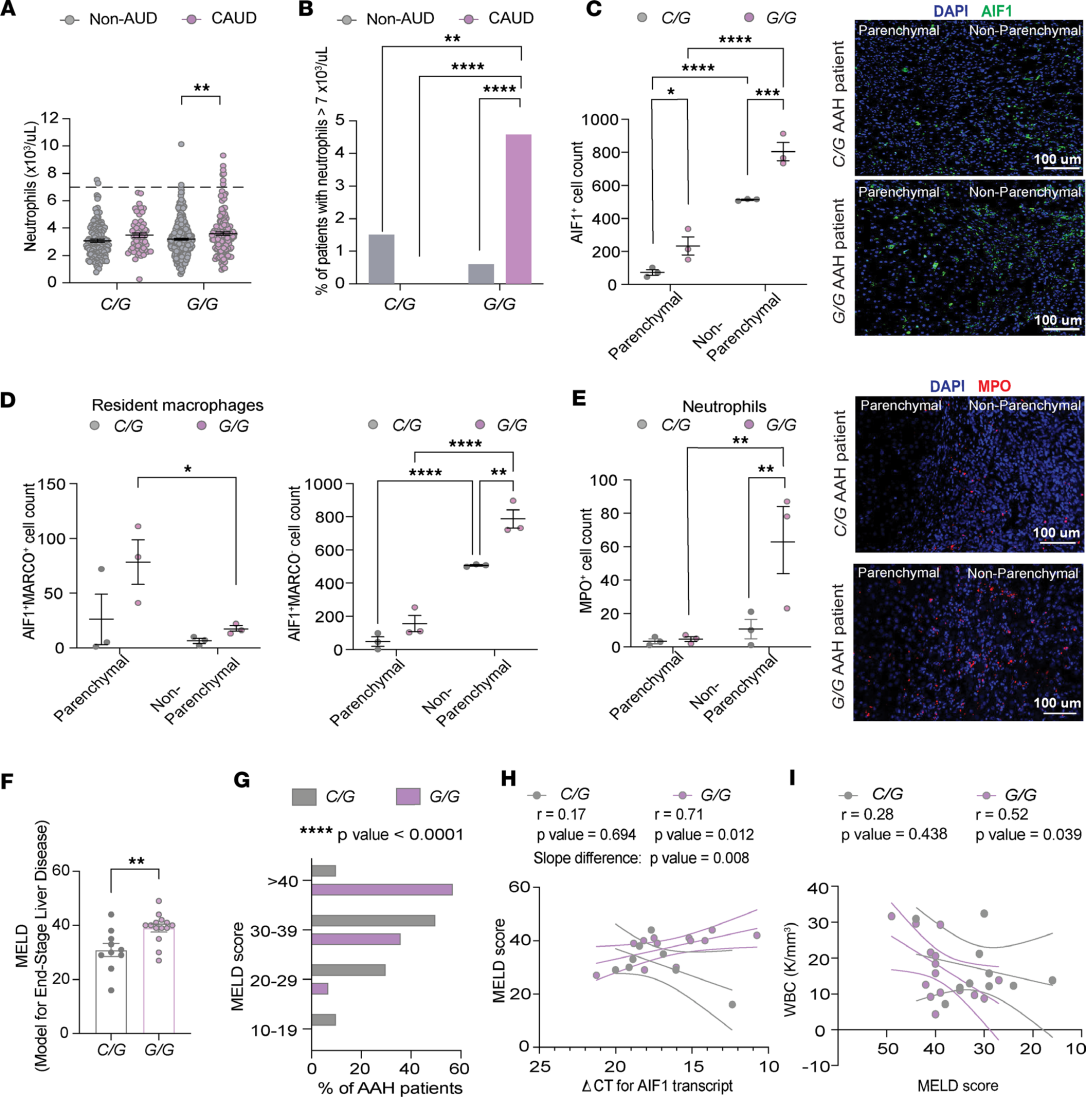
The rs3132451 *G/G* SNP is associated with elevated neutrophil counts in blood and liver and increased liver disease severity in ALD. (**A**) Scatter dot plot showing neutrophil levels in *C/G* and *G/G* patients within the non-AUD and CAUD groups. The dashed line indicates the upper limit of normal. Two-way ANOVA with Fisher’s LSD, **P = 0.0019. (**B**) Percentage of patients exceeding the upper limit of normal neutrophil levels by genotype and alcohol use. Binomial test, **P = 0.0087, ****P < 0.0001. (**C**) Left: Quantification of AIF1^+^ cells in liver tissue. Two-way ANOVA with Fisher’s LSD, *P < 0.05; ***P < 0.001; ****P < 0.0001. Right: Representative AIF1 immunostaining (green); DAPI (blue) nuclear counterstain. Scale bars: 100 μm. (**D**) Left: Quantification of liver AIF1^+^MARCO^+^ cells. Two-way ANOVA with Fisher’s LSD post hoc test, *P = 0.033. Right: Quantification of liver AIF1^+^MARCO^–^ cells, **P = 0.011, ****P < 0.0001. (**E**) Left: Quantification of MPO^+^ neutrophils in liver. Two-way ANOVA with Fisher’s LSD, **P < 0.01. Right: Representative MPO immunostaining (red); DAPI (blue) nuclear counterstain. Scale bars: 100 μm. (**F**) MELD scores in AAH patients by rs3132451 genotype. Mann-Whitney *U* test **P = 0.0074 and 2-tailed *t* test **P = 0.01. (**G**) MELD score distribution by genotype and percentage of affected AAH patients. χ^2^ test, ****P < 0.0001. (**H**) Linear regression of MELD score versus ΔCt values for AIF1 transcripts in liver samples from AAH patients relative to 18S, where lower ΔCt values indicate higher *AIF1* expression. Spearman’s *r* and *P* values, with the slope *P* value determined by *F* test. (**I**) Linear regression of MELD score versus serum white blood cell (WBC) count in AAH patients. Pearson’s *r* and *P* values are reported. In **C** and **E**, the image is 1 of 3 replicates from a single patient, representative of 3 patients analyzed. In **A**, **C**–**F**, **H**, and **I**, each point represents an individual, and data are shown as the mean ± SEM.

In healthy adults, neutrophil counts range from 1.5 × 10^3^ to 7 × 10^3^ cells/μL of blood (upper limit indicated by the dashed line in Figure 4A). A significantly higher percentage of *G/G* AUD patients had neutrophil counts exceeding 7 × 10^3^/μL, indicating neutrophilia (Figure 4B), a medical condition marked by abnormally high neutrophil levels. Elevated neutrophil counts in the blood, coupled with infiltration into the liver, are strongly linked to AAH, a particularly damaging form of ALD (47, 48). Thus, the *G/G* genotype may increase the likelihood of sustaining one of the key drivers of chronic inflammation.

Since the cohort of 868 participants did not include individuals with advanced ALD, the elevated serological markers of hepatic stress and injury observed in *G/G* individuals may reflect early manifestations of ALD, such as fatty liver. To further investigate the impact of the *AIF1* SNP on ALD, we analyzed a second cohort of 27 patients diagnosed with AAH. Liver biopsy tissue from each patient was genotyped at the rs3132451 locus, yielding 10 *C/G* and 17 *G/G* individuals. The duration of alcohol misuse did not differ significantly between *C/G* and *G/G* individuals (*C/G*, 22.24 ± 1.6 years; *G/G*, 20.2 ± 3.9 years; *P* = 0.582, 2-tailed *t* test). Similarly, there were no significant age differences between genotypes (*C/G*, 43.9 ± 2.8 years; *G/G*, 42.9 ± 2.1 years; *P* = 0.77).

Immunohistochemical staining for *AIF1* (also known as IBA1) in the liver revealed a 3-fold increase in AIF1^+^ cells within the parenchymal region and a 1.6-fold increase in the non-parenchymal area (Figure 4C) in *G/G* individuals. While the non-parenchymal compartment includes endothelial cells, stellate cells, portal fibroblasts, and various immune cell populations that interact during inflammatory liver injury, these cell types are not known to normally express AIF1/IBA1 (7). Likewise, hepatocytes, the predominant cell type in the liver parenchyma, do not express AIF1. In the liver, AIF1/IBA1 expression is largely restricted to Kupffer cells (resident macrophages) and infiltrating monocyte-derived macrophages (7).

As we observed a significant increase in AIF1^+^ cells in the liver (Figure 4C), we considered whether this reflected a general expansion of the macrophage population, through either infiltration of monocyte-derived macrophages or proliferation of resident Kupffer cells. To quantify these populations, we performed co-immunostaining for MARCO and AIF1 (Figure 4D). MARCO^+^AIF1^+^ cells represent classic Kupffer cells (resident macrophages), whereas MARCO^–^AIF1^+^ cells correspond to monocyte-derived macrophages recruited to the liver in response to injury, inflammation, infection, or fibrosis. Although MARCO^+^AIF1^+^ cell counts showed a non-significant trend toward higher numbers in both the parenchyma and non-parenchyma of *G/G* patients with severe ALD, they did not differ significantly from those in *C/G* patients (Figure 4D). Thus, the increase in total AIF1^+^ cells in *G/G* patients is more likely driven by expansion of MARCO^–^AIF1^+^ populations, consistent with infiltrating monocyte-derived macrophages. In support of this, *G/G* patients had significantly more MARCO^–^AIF1^+^ cells than *C/G* patients in the non-parenchymal region (Figure 4D).

Infiltrating monocyte-derived macrophages are blood-borne monocytes that enter the liver and differentiate in situ (4–6). When activated, these inflammatory macrophages promote a proinflammatory hepatic environment by secreting cytokines and chemoattractants that recruit neutrophils to sites of injury (14–17). In macrophages, AIF1 expression enhances these functions (10–13). To assess neutrophil recruitment, we quantified liver neutrophil levels using immunostaining for myeloperoxidase (MPO), a neutrophil granule protein (49). We observed a significantly higher number of neutrophils in non-parenchymal liver tissue of *G/G* patients (Figure 4E). Notably, hepatic sinusoids, which are part of the non-parenchymal vascular compartment, are known sites of neutrophil recruitment and accumulation during liver inflammation (50). While both *C/G* and *G/G* patients exhibited increased numbers of MARCO^–^AIF1^+^ cells in the non-parenchymal tissue compared with the parenchymal region (Figure 4D), likely reflecting the presence of infiltrating macrophages, only *G/G* patients showed elevated neutrophil counts in the non-parenchymal tissue. This suggests that individuals with the *G/G* genotype may be preferentially predisposed to neutrophil infiltration in AAH.

We also analyzed the MELD scores in *G/G* and *C/G* patients with AAH. The MELD score is a composite measure based on bilirubin, blood clotting time, and creatinine levels, used to assess liver function and the severity of a patient’s liver disease (51). The higher the MELD score, the more severe the liver dysfunction and the higher the risk of death (51). *G/G* patients had significantly higher MELD scores, with a median of 40 (IQR, 33.5–41) compared with 30.5 (IQR, 27.8–35.7) in *C/G* patients (Mann-Whitney *U* test, 2-tailed; Figure 4F), indicating an extremely high 90-day mortality risk (71.3%) and an urgent need for liver transplantation. Sex-stratified analysis revealed that both male and female *G/G* patients exhibited higher MELD scores compared with their *C/G* counterparts (Supplemental Figure 2). MELD score distribution analysis further supported increased disease severity in *G/G* patients (Figure 4G; χ^2^ test, *P* < 0.0001), with no significant differences in age across MELD score categories between genotypes (Supplemental Figure 2).

We also observed a strong correlation between liver *AIF1* transcript levels (ΔCt) and MELD score in *G/G* patients (Figure 4H), noting that lower ΔCt values correspond to higher *AIF1* expression. No correlation was observed in *C/G* patients (Figure 4H). Finally, we observed a moderate correlation between WBC counts and MELD score in *G/G* patients (Figure 4I), whereas no correlation was found in *C/G* patients (Figure 4I). Together, these findings suggest that *G/G* individuals have elevated circulating neutrophil levels in early-stage ALD and exhibit markedly increased hepatic neutrophil infiltration and disease severity in later stages. These results support a link between the *G/G* genotype at rs3132451 and a more severe ALD disease state, potentially driven by chronically elevated neutrophil levels.

### Males with CAUD exhibit greater increases in neutrophils, liver enzymes, and iron metabolism biomarkers.

Although the rs3132451 *G/G* SNP was associated with elevated neutrophils, AST, and iron levels at earlier, milder stages of liver damage — each of which may contribute to disease progression — we further investigated whether additional factors, such as sex, could influence the severity of AUD. While genetic variants that impact immune cell populations are important, prior studies have also highlighted sex differences in susceptibility to alcohol-related harm. Although males historically consume more alcohol and experience higher rates of alcohol-related injury and death (52), the gap between males and females has narrowed in recent years, prompting updated investigations into sex-specific effects. Using this contemporary dataset, we examined the impact of sex on study biomarkers in both AUD and non-AUD patients.

**Figure 5:**
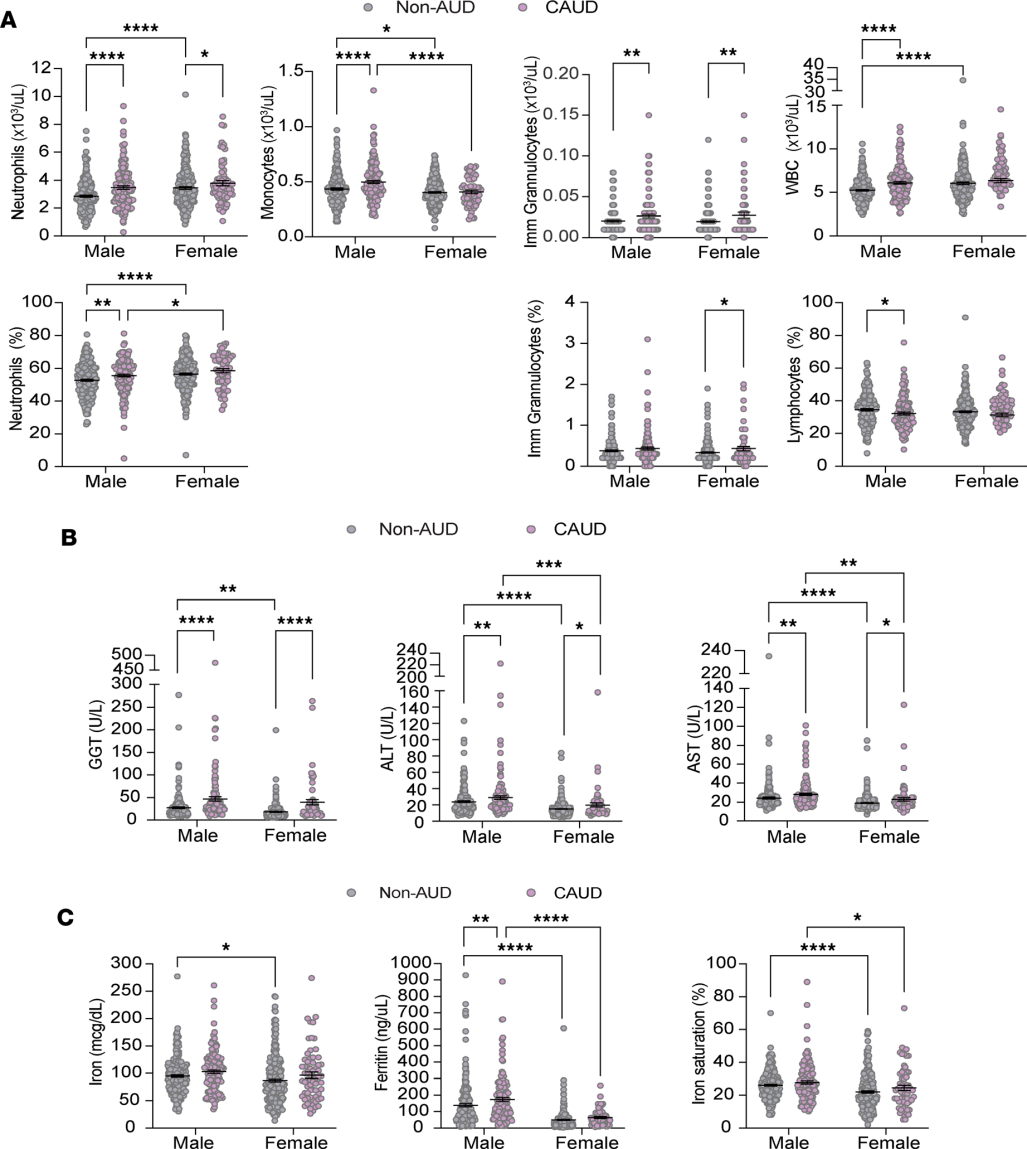
Males with CAUD exhibit greater increases in neutrophils, liver enzymes, and iron metabolism biomarkers. (**A**) Scatter dot plots showing immune cell populations altered by AUD analyzed across sexes. Imm, immature. (**B**) Same as in **A** but for liver function biomarkers. (**C**) Same as in **A** but for iron metabolism biomarkers. In **A**–**C**, each point represents an individual sample; data are shown as the mean ± SEM. Statistical comparisons for sex differences between the non-AUD and CAUD groups were assessed using a 2-way ANOVA, followed by Fisher’s LSD post hoc test for pairwise comparisons (α = 0.05). *P < 0.05, **P < 0.01, ***P < 0.001, ****P < 0.0001.

Our analysis recapitulated known sex differences in immune profiles: females generally had higher baseline neutrophil counts (53, 54), while males had higher monocyte levels (55) (Figure 5A). Chronic heavy alcohol use altered immune cell populations in both sexes — particularly immature granulocytes and neutrophils — but these effects were more pronounced in males (Figure 5A). Specifically, WBC count, monocytes, and neutrophil percentages were significantly elevated in male AUD patients compared with their non-AUD counterparts, while these changes were less apparent in females (Figure 5A). As shown in Figure 2A, AUD patients exhibited reduced lymphocyte percentages, with this reduction more striking in males — potentially compromising their antibody production and ability to fight bacterial infections.

Chronic heavy alcohol use significantly elevated GGT, ALT, and AST levels in both sexes (Figure 5B), but ALT and AST were notably higher in males with AUD. These increases likely reflect both alcohol’s hepatotoxicity and baseline sex differences, as males generally show higher levels of these liver enzymes (Figure 5B). Similarly, in the iron metabolism panel, males in the non-AUD group exhibited higher serum iron, ferritin, and transferrin saturation than females (Figure 5C), consistent with prior reports (56, 57). However, AUD males showed further increases in ferritin relative to non-AUD males, suggesting greater iron-related hepatic stress in this group.

For each dataset, we used 2-way ANOVA to estimate the independent contributions of genotype and sex to variability in immune and liver markers. Although not all parameters were measured in both the mild and severe ALD cohorts, we found that at the early stage of ALD, sex — rather than genotype — accounted for a larger proportion of total variability (Supplemental Table 2). By contrast, at the later stage of ALD, the contribution of genotype increased, in many cases exceeding the influence of sex (Supplemental Table 2). Together, these findings suggest that both genotype and sex shape susceptibility to alcohol-induced liver injury. In mild ALD, chronic alcohol exposure appears to affect immune function, liver enzymes, and iron metabolism more strongly in males than in females, with potential implications for clinical outcomes and recovery.

### The rs3132451 G/G genotype may hinder the normalization of neutrophil levels in males during alcohol abstinence.

Complete abstinence from alcohol is the cornerstone of both short-term and long-term survival in patients with ALD. However, managing liver disease decompensation remains a major challenge in treating ALD. Understanding whether certain genetic factors promote an easier path to AUD recovery with abstinence could provide insights for improving outcomes. In this study, we included a group of patients with AUD that did not meet DSM-IV/V criteria for AUD diagnosis for at least 1 year prior to data collection (LAUD patients) to investigate whether ceasing heavy, prolonged alcohol consumption can reverse the effects on enhanced immune function and liver damage. We also examined whether the rs3132451 SNP genotype or sex influences the reversal process.

**Figure 6:**
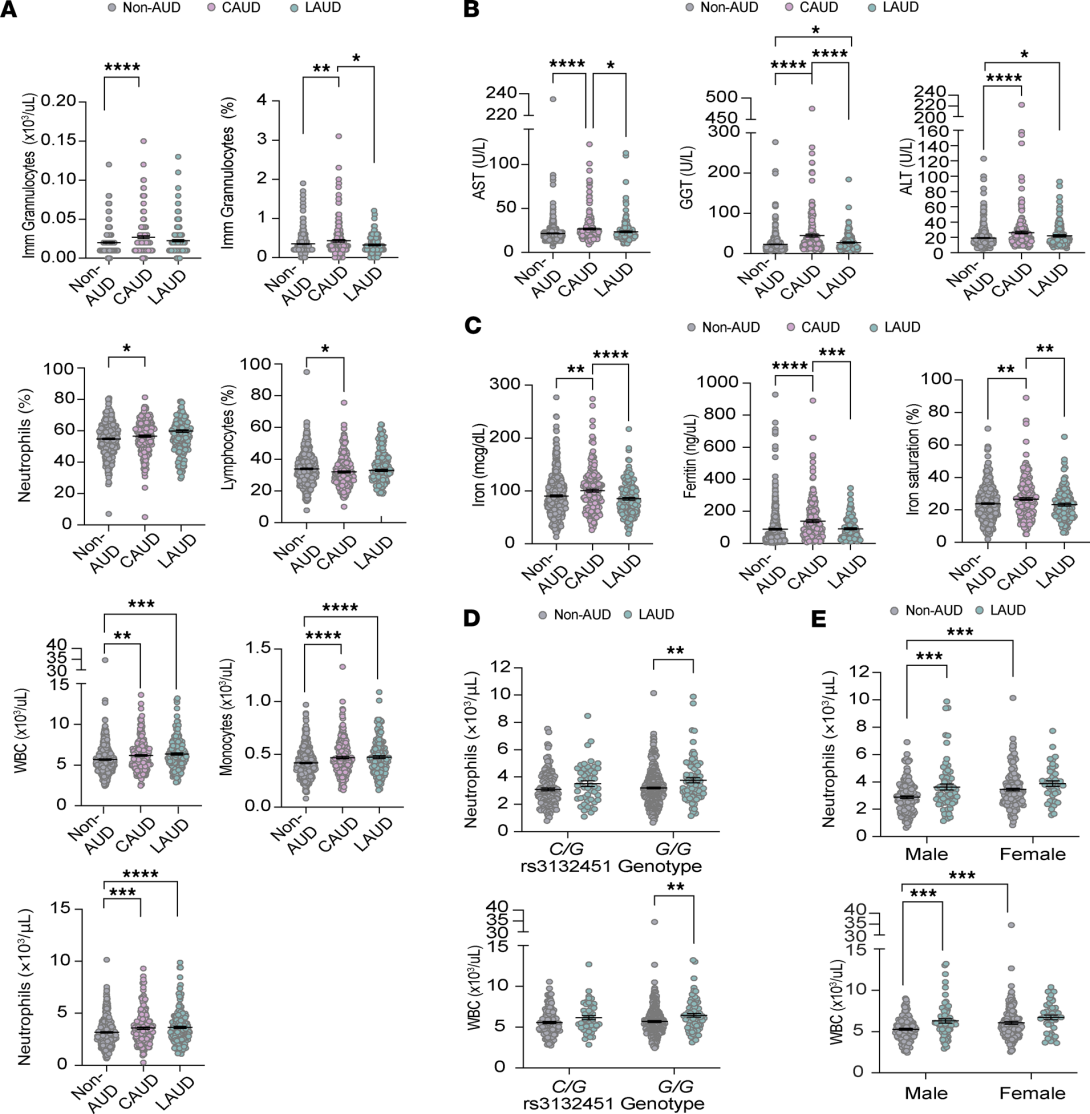
The rs3132451 *G/G* genotype may hinder the normalization of neutrophil levels in males during alcohol abstinence. (**A**) Immune cell populations — specifically granulocytes and lymphocytes — return to normal levels with alcohol abstinence; however, certain white blood cell (WBC) types, including monocytes and neutrophils, remain elevated. Elevated immune cell populations are observed in individuals with AUD. Statistical analysis was performed using unpaired 2-tailed Student’s *t* test (α = 0.05). Imm, immature. (**B**) Liver function biomarkers (AST, GGT, and ALT) normalize following alcohol abstinence. (**C**) Iron metabolism biomarkers return to normal levels with alcohol abstinence. (**D**) Scatter dot plots showing neutrophil and total WBC counts in individuals with *C/G* and *G/G* genotypes across the non-AUD and LAUD groups. (**E**) Scatter dot plots showing neutrophil and WBC counts in male and female individuals across the non-AUD and LAUD groups. In **A**–**E**, each point represents an individual sample; data are shown as the mean ± SEM. Statistical comparisons were made between individuals without a diagnosis of AUD (non-AUD), those with current AUD (CAUD; defined by DSM-IV/V criteria), and those with a lifetime history of AUD but not meeting criteria within the past year (LAUD), using unpaired 2-tailed Student’s *t* test (**A**–**C**) or 2-way ANOVA with Fisher’s LSD post hoc test (**D** and **E**) (α = 0.05). *P < 0.05, **P < 0.01, ***P < 0.001, ****P < 0.0001.

We analyzed the impact of alcohol use on 26 study biomarkers in patients with CAUD, no history of AUD (non-AUD), and LAUD. Normalization was observed in 4 immune-related biomarkers — absolute immature granulocyte count, immature granulocyte percentage, neutrophil percentage, and lymphocyte percentage — with alcohol abstinence (Figure 6A). However, overall WBC counts, particularly monocytes and neutrophils, remained elevated (Figure 6A). In contrast, biomarkers associated with liver injury and iron metabolism (both free and storage forms) that were elevated in CAUD patients normalized with alcohol abstinence (Figure 6, B and C). Altogether, these findings suggest that changes in immune cell populations may be among the most persistent effects of alcohol use, potentially requiring several years of abstinence to fully resolve.

Examining the effects of the rs3132451 SNP genotype and sex, we found that elevated neutrophil and WBC levels remained exclusively in the *G/G* patients with AUD compared with non-AUD patients (Figure 6D). Furthermore, prolonged increases in these cell types occurred more readily in males than females (Figure 6E). Overall, these results demonstrate that abstinence can reverse several adverse effects of chronic heavy alcohol consumption on liver enzymes, iron levels, and some immune cells. However, the recovery of neutrophil levels to the normal range may be influenced by both the rs3132451 genotype and sex.

### The rs3132451 G/G genotype effects are distinct to AUD and are not recapitulated in putative MASH subgroups.

In several figures, we note small absolute differences, though statistically significant, in the analyzed outcome measures (e.g., biomarkers of systemic iron accumulation, immune cell distributions, liver injury parameters) among the various subgroups within the large NAUD/CAUD cohort. In many cases, the *y* axis scale required to display all data points is quite large, which can visually diminish the apparent magnitude of these effects. Another possible contributing factor to these small absolute differences is the presence of undiagnosed metabolic dysfunction–associated steatohepatitis (MASH; formerly referred to as NASH) within the large non-AUD category. Using this cohort, we therefore examined how specific the rs3132451 SNP–associated phenotypes are to AUD, as compared with other forms of liver stress or injury (metabolic, infectious, etc.).

To explore this, we identified individuals in the non-AUD cohort who might meet criteria for MASH using 2 approaches: (a) a putative mild MASH group defined by ALT > AST and ALT > 40, and (b) a putative severe MASH group defined by AST > ALT and AST > 40.

MASH arises from metabolic dysfunction in which excess lipid delivery and impaired β-oxidation promote hepatic fat accumulation, de novo lipogenesis, mitochondrial dysfunction, and lipotoxicity (58). Immune activation in MASH is primarily driven by lipotoxicity and sterile inflammation. In contrast, AAH is primarily a toxin-mediated inflammatory condition. Thus, although both conditions share liver histopathology (steatosis, inflammation, hepatocyte ballooning, fibrosis), they differ substantially in their underlying mechanisms (58).

Across both MASH-enriched subsets, *G/G* individuals showed no statistically significant differences in any of the 26 biomarkers assessed, all related to immune activation, hepatic injury, or iron homeostasis (Supplemental Figures 3 and 4). However, in the putative severe MASH subset, there was a non-significant trend toward higher GGT, ALT, and AST levels in *G/G* patients compared with *C/G* patients (Supplemental Figure 4D). This trend raises the possibility that *G/G* individuals with confirmed severe MASH might exhibit significantly elevated liver enzymes and a more pronounced phenotype. Nonetheless, the absence of increases in immune cell parameters, liver injury markers, and iron-related indices among putative MASH individuals likely reflects the distinct primary mechanisms of AAH versus MASH. Taken together, these findings suggest that the rs3132451 SNP–associated phenotypes are more specific to AUD than to other forms of liver stress or injury.

## Discussion

In this study, we identified a genetic variant, rs3132451 (*C/C*, *C/G*, *G/G*), in the promoter region of the *AIF1* gene that predicts its expression. In CAUD, the *G/G* genotype is associated with elevated WBC counts, particularly neutrophils, in both the blood and liver, elevated serum AST levels, and increased susceptibility to iron overload. Males with CAUD are more severely affected, including during periods of alcohol abstinence, as the *G/G* genotype is associated with delayed normalization of WBC counts, particularly neutrophils.

The gene encoding AIF1or its homolog is expressed in invertebrate and vertebrate species — including sponges, fish, rodents, pigs, and humans — and shows strong conservation at the sequence level (59). From an evolutionary perspective, it is both evident and perplexing why the *G/G* genotype, which has the highest allelic frequency, could exert harmful effects. *AIF1* is highly expressed in phagocytic cells such as macrophages, Kupffer cells, and microglia, which engulf and destroy foreign particles like bacteria and viruses (12, 19, 47). In macrophages, *AIF1* has been shown to enhance their proliferation, migration, and activation (8, 10–13, 37, 38). In vitro experiments using the CRL-2192 macrophage cell line have shown that *AIF1* overexpression increases inducible nitric oxide synthase (iNOS) expression, nitric oxide (NO) production, and macrophage migration, whereas *AIF1* downregulation reduces iNOS expression and NO production and promotes apoptosis (13). These short-term elevations in *AIF1* expression may confer a selective advantage by enhancing immune responses and improving pathogen clearance, thereby increasing survival during infectious challenges. However, chronically elevated *AIF1* levels can be detrimental, as they may promote persistent, low-grade inflammation and heightened immune cell activity.

While disease-promoting alleles are often enriched in groups with more severe disease, this is not always the case. In this study, we did not find an enrichment of the *G/G* variant, which may reflect survival bias, whereby *G/G* homozygotes experience a higher likelihood of mortality, consistent with their significantly elevated MELD scores, leading to their underrepresentation among survivors with severe disease. This scenario represents a classic example of selection or collider bias, in which a disease-promoting allele would not necessarily appear more prevalent among liver transplant patients if *G/G* carriers (a) develop more severe alcohol-related disease and (b) die or are otherwise removed from the sampling frame before presentation or waitlisting. Consequently, the cross section of observed patients, such as clinic populations or transplant lists, could exhibit a lower *G/G* frequency than the general population or all individuals who ever developed severe disease. Faster disease progression and earlier mortality among *G/G* carriers could mask, or even reverse, the enrichment that might otherwise be expected.

In our cohort, *G/G* patients meet both criteria: they display more severe alcohol-related disease, evidenced by markedly elevated liver stress markers and a more than 3-fold increase in hepatic immune cells, particularly infiltrating AIF1^+^ macrophages and neutrophils, and they have substantially higher MELD scores (median 40), indicating increased mortality risk. By contrast, *C/G* and likely *C/C* patients exhibit milder disease and are less likely to die or be removed from the sampling frame before presentation or waitlisting, potentially leading to their overrepresentation among observed transplant candidates. Thus, although the *G/G* genotype has a strong disease-promoting effect, its enrichment may not be apparent among liver transplant recipients because of earlier mortality or accelerated disease progression prior to clinical observation.

As the increased susceptibility to neutrophilia was specific to the *G/G* genotype — and no CAUD patient with the *C/G* genotype exhibited similarly elevated neutrophil levels — this suggests that the combination of *AIF1* genetics and the CAUD condition has an additive effect on increasing circulating neutrophils. *AIF1* expression has been detected in low-density neutrophils (LDNs) and in peritoneal neutrophils during casein-induced inflammation (7, 12, 60). In LDNs, *AIF1* transcript levels reach approximately 600 transcripts per million (nTPM), compared with approximately 400–900 nTPM in monocytes (7, 60). Although *AIF1* is predominantly expressed in activated macrophages, its expression in circulating neutrophils may be restricted to specific subsets such as LDNs, particularly under inflammatory conditions. While the precise function of *AIF1* in neutrophils remains unclear, it is hypothesized — based on its role in macrophages — to support neutrophil activation and function (12).

Our findings in CAUD patients align with murine studies showing that 4 weeks of chronic intermittent ethanol vapor exposure increases circulating neutrophil counts and enhances their production of interferon-γ (IFN-γ) (61). *AIF1* expression is strongly induced by inflammatory stimuli like IFN-γ, further amplifying macrophage activity (12). Excess neutrophils may then release proinflammatory cytokines and chemokines, creating a feedback loop of immune activation. In *G/G* homozygotes, genetically driven upregulation of *AIF1* may exacerbate this inflammatory cascade. Although robust immune activation can be beneficial during infection, excessive neutrophil infiltration — especially in the context of chronic alcohol exposure, which independently promotes inflammation, cell death, and vulnerability to infection — can be detrimental (2, 5).

We also found that the *G/G* genotype is associated with an increased incidence of iron overload. In this study, iron was measured only in the blood, and we did not assess iron content within myeloid-lineage cells from *G/G* CAUD subjects. Therefore, we cannot determine how iron levels are altered in these cells, and in Figure 4, which focuses on myeloid lineage populations, we make no claims regarding intracellular iron content. Nevertheless, the *G/G* genotype is associated with greater serum iron overload and neutrophilia in both blood and liver. Investigating the relationship between iron status and myeloid lineage cells is an important direction for future studies.

Published literature indicates that iron plays a key role in neutrophil functions, including ROS production and phagocytosis. Experimental hypoferremia (low plasma iron) induced by hepcidin treatment in mice reduced ROS generation, reduced bactericidal activity against *Escherichia coli* and *Staphylococcus aureus*, and suppressed *CCL2* and *TNF-α* cytokine production in neutrophils following ex vivo stimulation with zymosan particles (26). In humans, iron deficiency anemia impairs the oxidative burst (27–29), whereas conditions characterized by iron overload, such as hereditary hemochromatosis (HH), enhance oxidative burst and phagocytic activity (29). HH patients also exhibit elevated levels of TNF-α, vascular cell adhesion molecule-1 (VCAM-1), and intercellular adhesion molecule-1 (ICAM-1), which may prime neutrophils for heightened activation (29, 62). Thus, *AIF1*-driven iron overload in CAUD may promote neutrophil hyperactivity, exacerbating tissue damage in an already inflamed liver.

Notably, male CAUD patients with the *G/G* genotype showed higher neutrophil counts, WBC levels, and ferritin concentrations and slower normalization during abstinence. Although both sexes possess the same immune cell types, men secrete more IL-6 and TNF-α from monocytes and macrophages (63), which can activate neutrophils, promote their migration, and contribute to tissue damage. Despite consuming less alcohol, women are generally more susceptible to ALD owing to factors like smaller body size, slower alcohol metabolism in the stomach, and heightened sensitivity of liver cells to oxidative stress. However, estrogen may offer some protection against immune-mediated damage. Estrogen, which is present at higher concentrations in women, dampens inflammation by suppressing FcγRIIIa — a receptor expressed on macrophages, mast cells, neutrophils, and NK cells — thereby modulating cytokine release, reducing monocyte production of IL-1β, IL-6, and TNF-α, and impairing neutrophil chemotaxis through decreased expression of iNOS and NO (64, 65). These sex-specific immune differences may explain the heightened vulnerability of male CAUD patients to *AIF1*-mediated immune dysregulation.

Fatty liver (alcohol-associated steatosis) can progress to more severe forms of ALD, including AAH and AC. Neutrophils are increasingly recognized as key mediators of ALD progression (66–68). AAH is characterized by substantial infiltration of monocyte-derived macrophages and neutrophils into the liver, driving inflammation damage (67, 68). Activated macrophages recruit neutrophils to the site of inflammation by releasing chemoattractants (e.g., CXCL1, CXCL2, IL-1α, CCL2) and survival factors (e.g., GM-CSF, G-CSF, TNF-α) that promote neutrophil recruitment and persistence, exacerbating hepatic injury (64, 68).

Although the precise mechanism by which *AIF1* influences neutrophil activity and hepatic iron levels in ALD progression remains unclear, targeting *AIF1* with small molecules, monoclonal antibodies, or gene-editing tools may hold therapeutic promise. Importantly, *AIF1*-knockout mice are viable, exhibit normal growth, and show reduced susceptibility to collagen-induced rheumatoid arthritis, an experimental model of chronic inflammation, suggesting that therapeutic inhibition of *AIF1* could reduce pathological immune activation without disrupting essential physiological functions (69). Moreover, identifying *AIF1* SNPs as risk factors may enable personalized treatment strategies for AUD and other *AIF1*-associated diseases, through genetic profiling and immune modulation.

### Limitations of the study

Several limitations of this study should be considered. First, the low frequency of the *C/C* genotype in the general population (estimated at about 2.3%) limited our ability to meaningfully assess the role of this genotype in AUD, liver function, and immune system integrity. Second, our cohort included more male than female participants with CAUD, reflecting established epidemiological trends wherein males exhibit higher rates of alcohol consumption and alcohol-related harm (52). Despite this imbalance, the distribution of *G/G* and *C/G* genotypes was similar between the non-AUD and AUD groups, and sex differences were observed in only 13 of the 26 biomarkers assessed. Third, there were approximately twice as many non-AUD individuals as CAUD patients in the study. Fourth, the AAH patient group was relatively small (*n* = 29), including only 7 female participants. Nonetheless, despite this limited sample size, we detected significant genotype-based differences in MELD scores among *C/G* and *G/G* individuals. Future studies should aim to include larger numbers of female and CAUD patients to enable more robust sex- and genotype-specific analyses.

Fifth, limited liver biopsy tissue availability restricted our ability to perform both histological and molecular analyses across all samples. Sixth, although we analyzed serum and urine samples from 868 individuals with and without CAUD, we did not assess macrophage populations in these fluids, as macrophages are typically absent from circulation in appreciable numbers. Consequently, our investigation of macrophage-related immune responses was confined to liver tissue, which was not available for non-AUD or CAUD individuals, given that liver biopsies are rarely performed in the absence of advanced liver disease. Lastly, while approximately 70% of individuals carry the *G/G* variant, the expressivity of *G/G*-linked phenotypes described here may be attenuated in some cases as a result of additional SNPs within the *AIF1* gene. These may act in *cis* or *trans* as missense mutations that reduce *AIF1* expression. As a result of such co-occurring mutations, the severity of health outcomes associated with the *G/G* variant may be underestimated.

## Methods

### Sex as a biological variable.

Both males and females were used in all human studies.

### Patients and sample collection.

The study included 868 participants from the NIH Clinical Center, ranging from healthy volunteers to those with AUD who were not seeking treatment. These participants were screened for research studies at the National Institute on Alcohol Abuse and Alcoholism (NIAAA) outpatient clinic. All participants completed the NIAAA Screening and Natural History Protocol, which allows for common assessments and broad eligibility criteria, enhancing the generalizability of the findings. Exclusion criteria were limited to individuals who were pregnant, breastfeeding, or under legal confinement. The participants included individuals without a diagnosis of AUD (non-AUD), those with current AUD (CAUD; based on the criteria for alcohol abuse and alcohol dependence outlined in the fourth and fifth editions of the Diagnostic and Statistical Manual of Mental Disorders [DSM-IV/V]), and those with a lifetime diagnosis of AUD but not in the year preceding sample collection (long-term abstinent AUD [LAUD]). Patients underwent blood and urine tests.

Liver tissue and blood samples were obtained from patients diagnosed with acute alcohol-associated hepatitis who were evaluated for liver transplantation at Johns Hopkins Hospital (70).

Epstein-Barr virus–immortalized (EBV-immortalized) lymphoblastoid cell lines were prepared from samples previously described elsewhere (71).

### Genotyping.

Genomic DNA was extracted using TRIzol (Invitrogen) according to the manufacturer’s protocol and dispensed into 384-well plates, dried, and stored at room temperature. Genotyping of human DNA samples was performed using validated TaqMan SNP Genotyping Assays rs3132451 (assay ID: C___2451891_10, Thermo Fisher Scientific) and rs2736182 (assay ID: C__16286882_10, Thermo Fisher Scientific). Each reaction was carried out in a final volume of 5 μL, composed of 2.5 μL of TaqMan Universal PCR Master Mix, 0.125 μL of the allele-specific assay mix, 10 ng of genomic DNA, and nuclease-free water to reach the total volume. Thermal cycling was conducted using a PCR system beginning with an initial denaturation at 95°C for 10 minutes, followed by 40 cycles of denaturation at 95°C for 15 seconds and annealing/extension at 60°C for 1 minute. Fluorescence signals from VIC- and FAM-labeled probes were measured using a real-time PCR system (QuantStudio 7, Applied Biosystems) to distinguish between alleles. Genotype calling was performed automatically using the QuantStudio 7 software.

### Real-time quantitative PCR analysis.

EBV-immortalized lymphoblastoid cell lines were lysed in TRIzol (Invitrogen), and total RNA was isolated according to the manufacturer’s instructions. Total RNA was purified using the RNeasy Mini Kit (QIAGEN) and RNase-free DNase set (QIAGEN) following the manufacturer’s protocol. RNA was eluted in RNase-free water and quantified using a NanoDrop spectrophotometer. First-strand cDNA was synthesized using the SuperScript VILO Master Mix (Thermo Fisher Scientific) following the standard procedure recommended by the manufacturer. Gene expression levels were measured using TaqMan Gene Expression Assays rs3132451 (FAM dye labeled; assay ID: Hs00610419_g, Thermo Fisher Scientific). Each reaction was carried out in a final volume of 5 μL, containing 2.5 μL of TaqMan Universal PCR Master Mix, 0.25 μL of each specific TaqMan assay, and 2 μL of cDNA template. Amplification was performed on a real-time PCR system (QuantStudio 7, Applied Biosystems) under the following conditions: an initial denaturation step at 95°C for 20 seconds, followed by 40 cycles of 95°C for 1 second and 60°C for 20 seconds. All reactions were run in technical triplicates, and gene expression levels were adjusted relative to the housekeeping gene *ACTB* using the comparative Ct method (ΔCt = Ct*_AIF1_* – Ct*_ACTB_*). EBV-immortalized lymphoblastoid cell lines were derived from African American patients, who exhibit a high frequency of the rs2736182 SNP (G>A, chr6:31615535, African American allele frequency = 0.1994, gnomAD [v4, Broad Institute]) (31). This SNP interferes with probe hybridization in TaqMan Gene Expression Assays (rs3132451, chr6:31615209–31617025). Consequently, all samples with *A/G* or *A/A* genotypes at rs2736182 (25 of 72) were excluded from the analysis.

### Immunofluorescent staining.

Formalin-fixed, paraffin-embedded liver sections of 5 mm thickness were deparaffinized and rehydrated by passage through xylene (3 washes) and graded series of ethanol (100%, 95%, 70%; each with one wash). Then the sections were permeabilized using 0.3% Triton X-100 (in 1× PBS) for 10 minutes at room temperature. Antigen was retrieved by either citrate buffer (pH 6.0) or heating of the sections in a microwave oven in antigen retrieval reagent (Enzo Life Sciences). Endogenous peroxidase activity was quenched by incubation of the sections in 3% H_2_O_2_ (in 1× PBS) at room temperature. The sections were then blocked with 10% normal goat serum for 1 hour at room temperature and incubated with anti-MARCO antibody (reference PA5-64134; Invitrogen), anti-MPO antibody (catalog CPTC-MPO-1; Developmental Studies Hybridoma Bank), or anti-*AIF1* antibody (catalog MABN92; Sigma-Aldrich) overnight at 4°C. The following day, sections were incubated with fluorescent-labeled secondary antibodies at room temperature for 1 hour, and the nuclear material was stained with DAPI Fluoromount-G (Electron Microscopy Sciences). The sections were coverslipped and imaged under a bright-field fluorescence slide scanner (Aperio VERSA Slide Scanner 8, Leica Biosystems, USA) at ×20 magnification.

### Cell count quantification.

MPO^+^ and *AIF1^+^* cell quantification was performed using QuPath (72) and ImageJ software (73). Analysis of liver tissue images in QuPath involved selecting 500 μm × 500 μm regions from both parenchymal and non-parenchymal areas. In ImageJ, cell counts were obtained by assessment of colocalization between DAPI-stained nuclei and red/green fluorescence marking MPO or AIF1 expression. After color thresholding, images were binarized, converted to masks, and processed using the watershed algorithm to separate overlapping cells. The Analyze Particles function was used to count cells with a minimum diameter threshold of 3.2 μm. Each region was quantified in triplicate, blindly to genotype. MARCO^+^ cells were identified following the same protocol; however, only cells larger than 15 μm and coexpressing *AIF1* were included in the analysis.

### Statistics.

Statistical analyses were conducted to evaluate differences across experimental groups. The number of individuals per group is denoted as *n*. Based on the sample size, as supported by the central limit theorem, the distribution of biomarker data was assumed to be approximately normal. Values are presented as the mean ± SEM. Comparisons between categorical variables were performed using the binomial test, Fisher’s exact test, or χ^2^ test, depending on expected frequencies and number of groups compared. Unpaired 2-tailed Student’s *t* tests were used for 2-group comparisons, and a 2-way ANOVA was applied for comparisons involving the interaction of 2 variables, whereas a 1-way ANOVA was used for single-variable comparisons. Post hoc analyses following ANOVA were performed using Fisher’s least significant difference (LSD) test without correction for multiple comparisons. Pearson’s correlation analysis was conducted to evaluate the linear relationship between pairs of variables, with the strength of association indicated by the Pearson’s *r* and *P* value. Spearman’s rank correlation was additionally performed to evaluate monotonic associations. To compare regression lines between groups, analysis of covariance (ANCOVA) was performed. This included an *F* test to assess the interaction between group and predictor variables, thereby determining whether the slopes of the regression lines differed significantly. All statistical analyses were performed using GraphPad Prism. Results were considered statistically significant at *P* < 0.05. Full statistical details are provided in the relevant figure panels and legends.

### Study approval.

All patients from NIH Clinical Center provided written informed consent in accordance with an NIH institutional review board–approved (IRB-approved) protocol, IRB protocol 14-AA-0181. Lymphoblast donors gave written informed consent to the study that was approved by the IRBs of the Department of Veterans Affairs New Jersey Healthcare System (East Orange, NJ) and the University of Medicine and Dentistry, New Jersey Medical School at Newark. Informed consent was obtained for the use, sharing, and publication of research findings derived from AAH patient specimens, as approved by the Johns Hopkins Hospital IRB, IRB00107893.

### Data availability.

Controlled public access of phenotype and genotype data from the NIAAA Natural History Protocol can be made through the National Institute of Mental Health Data Archive, record 5072 (https://www.niaaa.nih.gov/research/data-archive-and-resources/niaaa-data-archive). Values for all data points in graphs are reported in the Supporting Data Values file.

## Author contributions

MWA, PCA, and DG conceived and designed the study. MWA and PCA wrote the manuscript. MWA, DG, ZS, and BG provided resources. CAH and MAP performed data acquisition, and CAH conducted data preprocessing. PCA and MWA analyzed and interpreted the data and conducted the statistical analysis. CAH designed the quantitative reverse transcription PCR experiments; PCA, CM, and MWA performed RNA and DNA extraction, amplification, and analysis. PCA, DF, MWA, and BMK performed tissue processing, imaging, and cell quantification.

## Conflict of interest

The authors have declared that no conflict of interest exists.

## Funding support

This work is the result of NIH funding, in whole or in part, and is subject to the NIH Public Access Policy. Through acceptance of this federal funding, the NIH has been given a right to make the work publicly available in PubMed Central.

- NIH Intramural Research Program grant 1ZIAAA000440-02 to MWA.
- NIH grant R24AA025017 to the Transplant Biology Research Center of Johns Hopkins University School of Medicine.

## Supplementary Material

Supplemental data

ICMJE disclosure forms

Supporting data values

## Figures and Tables

**Table 1:**
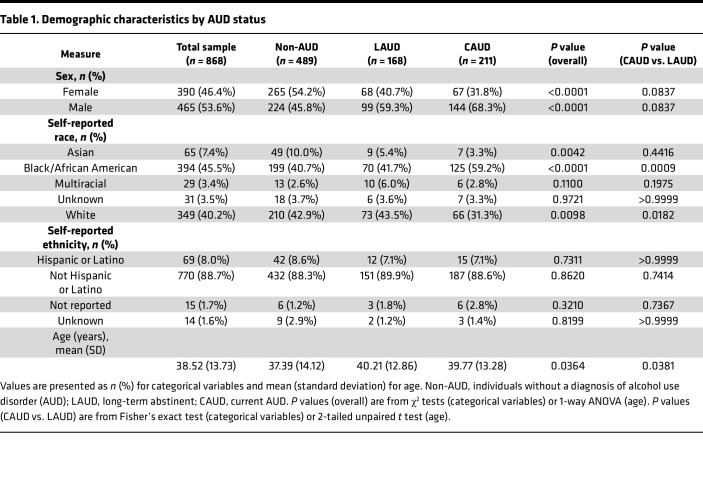
Demographic characteristics by AUD status

## Version 1 03/16/2026

In-Press Preview

## Version 2 04/22/2026

Electronic publication
