# Supplementary material for: Genetic regulation of AIF1 shapes immune and liver injury profiles in chronic alcohol use
Source: JCI Insight. 2026 Mar 10;11(8):e198209. doi: 10.1172/jci.insight.198209 (PMC13135398; doi:10.1172/jci.insight.198209)
Supplement: Supplemental data [file jciinsight-11-198209-s182.pdf]

Supplemental Table 1: Observed AIF1 rs3132451 genotype frequencies fall within expected population ranges in the severe alcohol-associated hepatitis cohort

Genotype	Severe ALD (n = 29)	Non-AUD (n = 489)		CAUD (n = 211)		LAUD (n = 168)	
	n (%)	*n* (%)	*p-value*	*n* (%)	*p-value*	*n* (%)	*p-value*
C/C	2 (6.9%)	11 (2.25%)	0.140	3 (1.42%)	0.062	6 (3.57%)	0.280
C/G	10 (34.5%)	134 (27.40%)	0.407	55 (26.07%)	0.295	48 (28.57%)	0.538
G/G	17 (58.6%)	344 (70.35%)	0.221	153 (72.51%)	0.099	114 (67.86%)	0.321

Genotype	Severe ALD (n = 29)	Average in general population		Chi-square test
	n (%)	%	*p-value*	Severe ALD vs non-AUD: p=0.1836
C/C	2 (6.9%)	2.3 %	0.1431	Severe ALD vs CAUD: p=0.0809
C/G	10 (34.5%)	22.6 %	0.1252	Severe ALD vs LAUD: p=0.5262
G/G	17 (58.6%)	75.1 %	0.0516	Severe ALD vs Average in general population: p=0.0739

Values are presented as n (%). Genotype frequencies in the severe alcohol-associated liver disease (ALD) cohort (n = 29) were compared with individuals without a diagnosis of alcohol use disorder (Non-AUD; n = 489), with current AUD (CAUD, per DSM-IV/V criteria; n = 211), and with a lifetime history of AUD but not meeting criteria within the past year (LAUD; n = 168) cohorts. P values reflect comparisons between the severe ALD group using a two-tailed binomial test. The genotype distribution in severe ALD was compared with other cohorts using the chi-square test, with the corresponding p-values reported.

Supplemental Table 2: Percentage of total variation from ANOVA analyses of immune and liver markers in CAUD individuals by genotype and sex.

Early stage, Steatosis probable

	Sex	Genotype	Interaction
WBCs	0.1148	0.013	0.089
AST	2.177	0.673	0.031
ALT	1.663	0.143	0.046
Ferritin	10.56	0.300	0.067
Transferrin	6.755	0.716	0.202
Iron	0.6496	0.0433	0.283

Liver Biopsy and Listed MELD are not applicable for cases of mild liver disease

Late stage, Alcoholic Hepatitis diagnosed

	Sex	Genotype	Interaction
WBCs	3.175	0.012	3.712
AST	13.17	20.47	8.764
ALT	2.361	2.664	0.3647

Ferritin, Transferrin, Iron not measured

	Sex	Genotype	Interaction	*Liver area	Genotype	Interaction
Listed MELD	0.006	20.86	0.6678			
AIF1 liver transcript levels (ΔCT)	6.325	8.236	3.702			
$AIF1^{+}$ cells in the liver				79.01	15.75	1.324
Neutrophils (MPO^{+}) cells in the liver				32.38	21.58	19.47

Samples classified by *liver area (parenchyma vs. non-parenchymal) did not have sufficient numbers for analysis by sex.

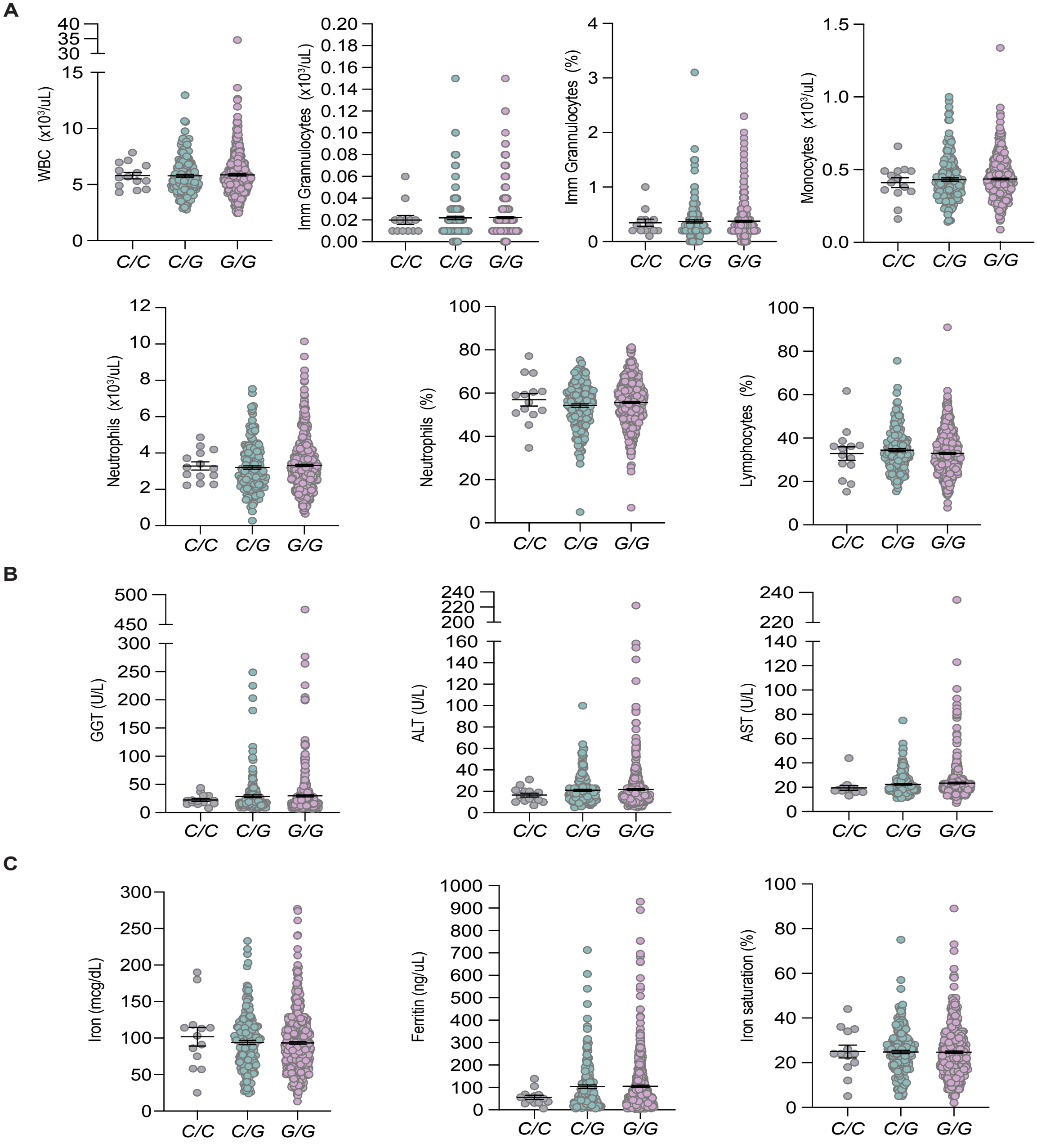
A
WBC (x10³/uL)
Imm Granulocytes (x10³/uL)
Imm Grannulocytes (%)
Monocytes (x10³/uL)
Neutrophils (x10³/uL)
Neutrophils (%)
Lymphocytes (%)
B
GGT (U/L)
ALT (U/L)
AST (U/L)
C
Iron (mcg/dL)
Ferritin (ng/uL)
Iron saturation (%)
C/C
C/G
G/G

Supplemental Figure 1. No genotype-dependent differences in immune, liver Injury, or iron biomarkers.
(A) Scatter dot plots showing immune cell populations stratified by rs3132451 genotypes (*C/C*, *C/G*, *G/G*).
(B) Same as (A), but for iron levels and biomarkers of iron metabolism.
(C) Same as (A), but for liver function biomarkers.
Each point represents an individual patient; bars indicate the mean ± standard error of the mean. Statistical comparisons were conducted using one-way ANOVA with Tukey's post hoc test ($\alpha = 0.05$). All p-values were > 0.05.

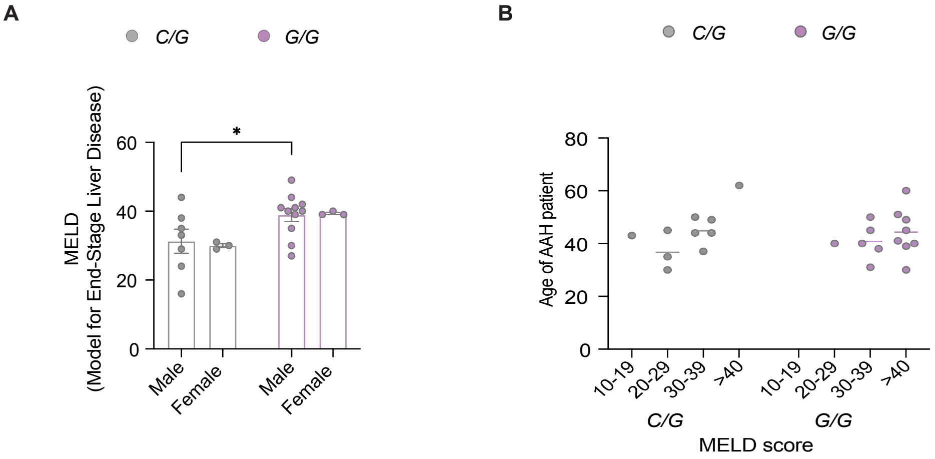

Supplemental Figure 2. Elevated MELD scores in male patients with Alcohol-Associated Hepatitis(AAH) carrying the G/G genotype, with no detectable genotype-dependent differences in the age-related distribution of MELD.

(A) MELD scores stratified by sex and genotype. Two-way ANOVA with Fisher's LSD post hoc test: *p = 0.0294 (males).

(B) MELD scores by genotype and age in AAH patients.

In **A-B**, each point represents an individual patient; bars indicate the mean ± standard error of the mean.

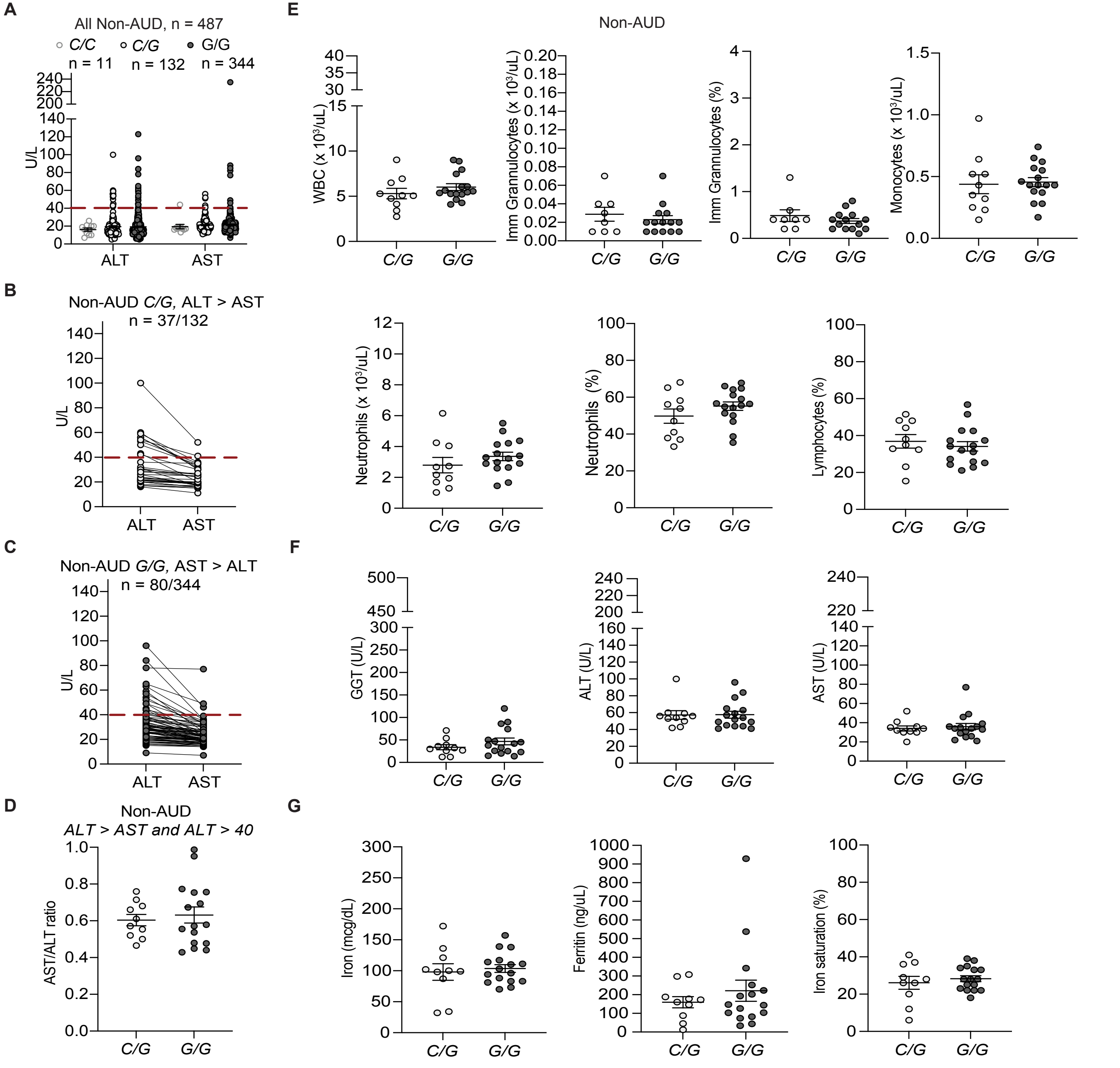
A
All Non-AUD, n = 487
C/C n = 11
C/G n = 132
G/G n = 344
U/L
ALT
AST
B
Non-AUD C/G, ALT > AST
n = 37/132
U/L
ALT
AST
C
Non-AUD G/G, AST > ALT
n = 80/344
U/L
ALT
AST
D
Non-AUD
ALT > AST and ALT > 40
AST/ALT ratio
C/G
G/G
E
Non-AUD
WBC (x 10³/uL)
Imm Grannulocytes (x 10³/uL)
Imm Grannulocytes (%)
Monocytes (x 10³/uL)
Neutrophils (x 10³/uL)
Neutrophils (%)
Lymphocytes (%)
C/G
G/G
F
GGT (U/L)
ALT (U/L)
AST (U/L)
G
Iron (mcg/dL)
Ferritin (ng/uL)
Iron saturation (%)

Supplemental Figure 3. G/G non-AUD individuals with putative mild metabolic dysfunction associated steatohepatitis (MASH) show no differences in the levels of immune, liver-injury, or iron biomarkers.

(A) Scatter dot plots showing ALT and AST levels in all non-AUD individuals in the study (n=487) stratified by rs3132451 genotypes (C/C, C/G, G/G). The dashed line denotes the upper limit of normal for ALT i.e. > 40.

(B) Paired ALT and AST levels for C/G patients. Only 37/132 patients had ALT levels higher than AST (ALT > AST), and of these, only 10 met the criterion for putative mild MASH (ALT > AST and ALT > 40). Note, no C/C individuals met these criteria.

(C) Paired ALT and AST levels for G/G patients. Only 80/344 patients had ALT levels higher than AST (ALT > AST), and of these, only 16 met the criterion for putative mild MASH (ALT > AST and ALT > 40). Note, no C/C individuals met these criteria.

(D) The AST/ALT ratio did not differ between C/G and G/G individuals meeting criteria for putative mild MASH.

(E) Scatter dot plots of immune cell populations for C/G and G/G individuals meeting criteria.

(F) Same as (E), for liver function biomarkers.

(G) Same as (E), for iron levels and iron-related biomarkers.

In **A-G**, each point represents an individual; bars show mean ± SEM. Statistical comparisons were performed with unpaired two-tailed Student's t-tests ($\alpha = 0.05$); all p-values were > 0.05.

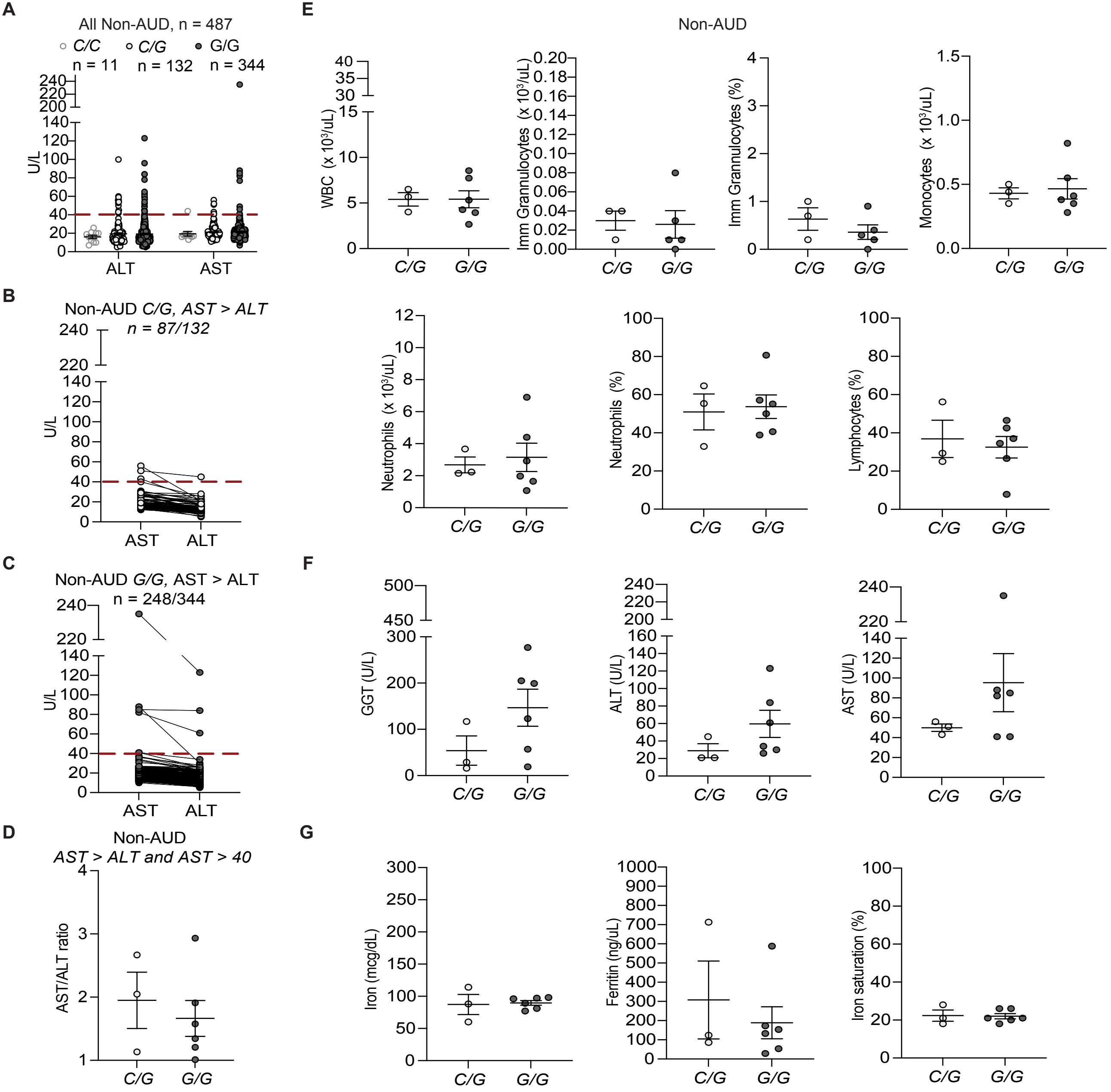
A
All Non-AUD, n = 487
C/C
C/G
G/G
n = 11
n = 132
n = 344
U/L
ALT
AST
B
Non-AUD C/G, AST > ALT
n = 87/132
U/L
AST
ALT
C
Non-AUD G/G, AST > ALT
n = 248/344
U/L
AST
ALT
D
Non-AUD
AST > ALT and AST > 40
AST/ALT ratio
C/G
G/G
E
Non-AUD
WBC (x 10³/uL)
Imm Granulocytes (x 10³/uL)
Imm Granulocytes (%)
Monocytes (x 10³/uL)
Neutrophils (x 10³/uL)
Neutrophils (%)
Lymphocytes (%)
C/G
G/G
F
GGT (U/L)
ALT (U/L)
AST (U/L)
C/G
G/G
G
Iron (mcg/dL)
Ferritin (ng/uL)
Iron saturation (%)
C/G
G/G

Supplemental Figure 4. ***G/G*** **non-AUD individuals with putative severe metabolic dysfunction associated steatohepatitis (MASH) show no differences in the levels of immune, liver-injury, or iron biomarkers.**

(A) Scatter dot plots showing ALT and AST levels in all non-AUD individuals in the study (n=487) stratified by rs3132451 genotypes (*C/C*, *C/G*, *G/G*). The dashed line denotes the upper limit of normal for AST i.e. > 40.

(B) Paired AST and ALT levels for *C/G* patients. Only 87/132 patients had AST levels higher than ALT (AST > ALT), and of these, only 3 met the criterion for putative severe MASH (AST > ALT and AST > 40). Note, no *C/C* individuals met these criteria.

(C) Paired AST and ALT levels for *G/G* patients. Only 248/344 patients had AST levels higher than ALT (AST > ALT), and of these, only 6 met the criterion for putative severe MASH (AST > ALT and AST > 40). Note, no *C/C* individuals met these criteria.

(D) The AST/ALT ratio did not differ between *C/G* and *G/G* individuals meeting criteria for putative severe MASH.

(E) Scatter dot plots of immune cell populations for *C/G* and *G/G* individuals meeting criteria.

(F) Same as (E), for liver function biomarkers.

(G) Same as (E), for iron levels and iron-related biomarkers.

In **A-G**, each point represents an individual; bars show mean ± SEM. Statistical comparisons were performed with unpaired two-tailed Student's t-tests ($\alpha = 0.05$); all p-values were > 0.05.
